# Supplementary material for: Unique Polyhalogenated Peptides from the Marine Sponge Ircinia sp
Source: Mar Drugs. 2020 Jul 28;18(8):396. doi: 10.3390/md18080396 (PMC7460063; doi:10.3390/md18080396)
Supplement: Supplementary file 1 [file marinedrugs-18-00396-s001.pdf]

Unique Polyhalogenated Peptides from the Marine Sponge *Ircinia* sp.

Rogelio Fernández ID,[3] Asep Bayu,[1] Tri Aryono Hadi,[2] Santiago Bueno,[3] Marta Pérez ID,[3,*] Carmen Cuevas[3] and Masteria Yunovilsa Putra[1]

[1] Research Center for Biotechnology, Indonesian Institute of Sciences, Jl. Raya Jakarta-Bogor No.Km46, Cibinong, Bogor, Jawa Barat 16911

[2] Research Center for Oceanography, Indonesian Institute of Sciences, Jl. Pasir Putih I, Ancol Timur, Jakarta 14430

[3] Natural Products Department, PharmaMar S.A., Pol. Ind. La Mina Norte, Avda. de los Reyes 1, 28770 Colmenar Viejo (Madrid), Spain.; mperez@pharmamar.com

Experimental Procedure

Figure S1. Picture of the fresh sponge.
Figure S2. ^{1}H NMR spectrum of Haloircinamide A (**1**) 500 MHz, DMSO.
Figure S3. ^{13}C NMR spectrum of Haloircinamide A (**1**) 125 MHz, DMSO.
Figure S4. g-HSQC spectrum of Haloircinamide A (**1**) 500 MHz, DMSO.
Figure S5. g-COSY spectrum of Haloircinamide A (**1**) 500 MHz, DMSO.
Figure S6. TOCSY spectrum of Haloircinamide A (**1**) 500 MHz, DMSO.
Figure S7. g-HMBC spectrum of Haloircinamide A (**1**) 500 MHz, DMSO.
Figure S8. ^{1}H NMR spectrum of Haloircinamide A (**1**) 500 MHz, CD_3OD.
Figure S9. ^{13}C NMR spectrum of Haloircinamide A (**1**) 125 MHz, CD_3O).
Figure S10. g-HSQC spectrum of Haloircinamide A (**1**) 500 MHz, CD_3OD.
Figure S11. g-COSY spectrum of Haloircinamide A (**1**) 500 MHz, CD_3OD.
Figure S12. g-HMBC spectrum of Haloircinamide A (**1**) 500 MHz, CD3OD.
Figure S13. ^{1}H NMR spectrum of Haloircinamide A (**1**) 500 MHz, CD_3OH.
Figure S14. g-COSY spectrum of Haloircinamide A (**1**) 500 MHz, CD_3OH.
Figure S15. g-HMBC spectrum of Haloircinamide A (**1**) 500 MHz, CD_3OH.
Figure S16. ROESY spectrum of Haloircinamide A (**1**) 500 MHz, CD3OH.
Figure S17. ^{1}H NMR spectrum of Seribunamide A (**2**) 500 MHz, DMSO.
Figure S18. ^{13}C NMR spectrum of Seribunamide A (**2**) 125 MHz, DMS).
Figure S19. g-HSQC spectrum of Seribunamide A (**2**) 500 MHz, DMSO.
Figure S20. g-COSY spectrum of Seribunamide A (**2**) 500 MHz, DMSO.
Figure S21. g-HMBC spectrum of Seribunamide A (**2**) 500 MHz, DMSO.
Figure S22. ROESY spectrum of Seribunamide A (**2**) 500 MHz, DMSO.
Figure S23. ^{1}H NMR spectrum of Seribunamide A (**2**) 500 MHz, CD_3OD.
Figure S24. ^{13}C NMR spectrum of Seribunamide A (**2**) 125 MHz, CD_3OD.
Figure S25. g-HSQC spectrum of Seribunamide A (**2**) 500 MHz, CD_3OD.
Figure S26. g-COSY spectrum of Seribunamide A (**2**) 500 MHz, CD_3OD.
Figure S27. TOCSY spectrum of Seribunamide A (**2**) 500 MHz, CD3OD.
Figure S28. g-HMBC spectrum of Seribunamide A (**2**) 500 MHz, CD3OD.
Figure S29. ROESY spectrum of Seribunamide A (**2**) 500 MHz, CD3OD.
Figure S30. QTOFMS spectrum of Haloirciamide A (**1**) and fragments found
Figure S31. Marfey of Haloirciamide A (**1**) and iSer standards
Figure S32. Marfey of Haloirciamide A (**1**) and Asp standards
Figure S33. Marfey of Haloirciamide A (**1**) and Dap standards

Figure S34. Marfey of Seribunamide A (**2**) and Ile standards
Figure S35. Marfey of Seribunamide A (**2**)
Figure S36. Marfey of Seribunamide A (**2**). Pro standards
Figure S37. Marfey of Seribunamide A (**2**). NMeLeu standards
Figure S38. Marfey of Seribunamide A (**2**). Glu standards

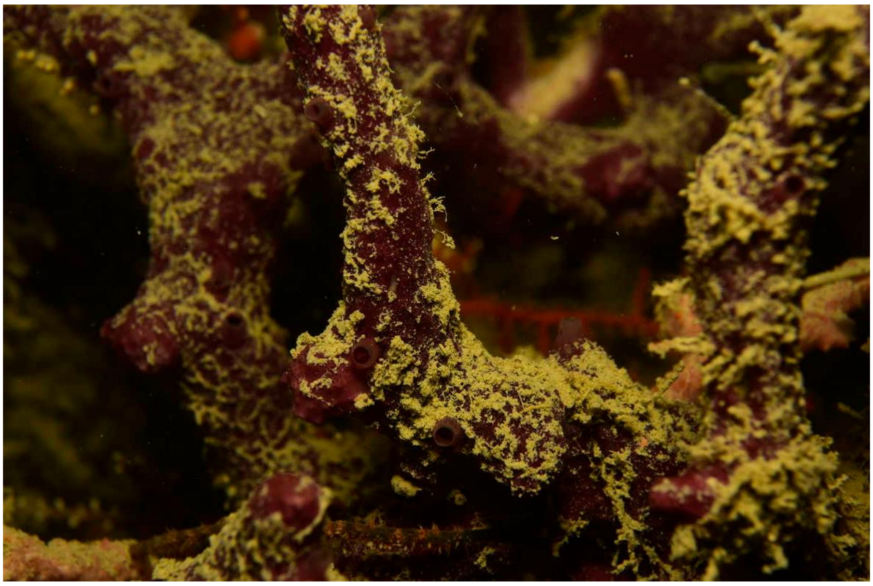

Figure S1. Picture of the fresh sponge.

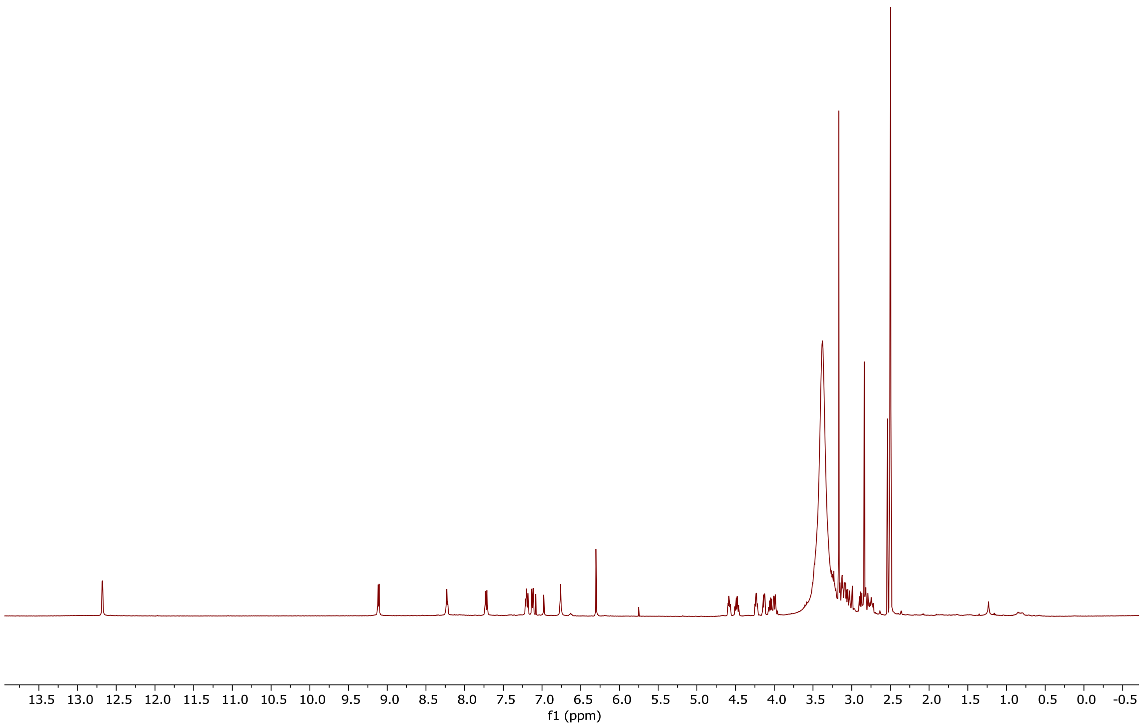

Figure S2 ^{1}H NMR spectrum of Haloircinamide A (**1**) 500 MHz, DMSO.

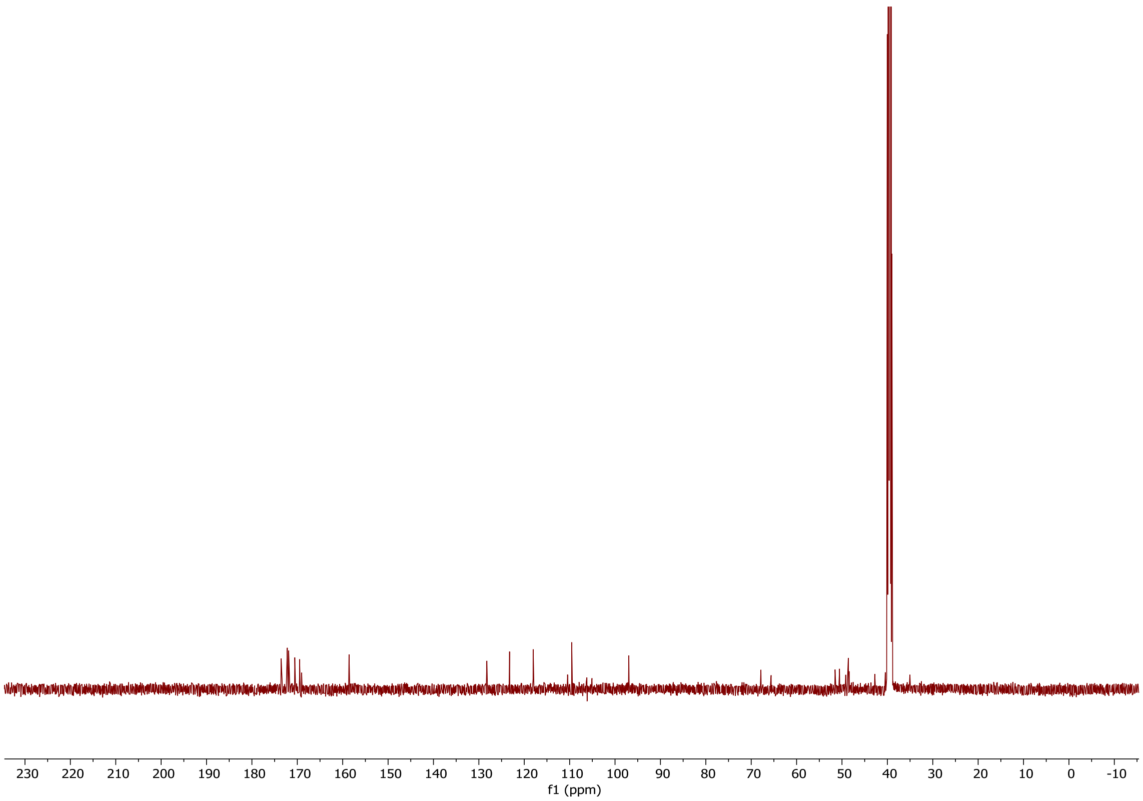

Figure S3 ^{13}C NMR spectrum of Haloircinamide A (**1**) 125 MHz, DMSO.

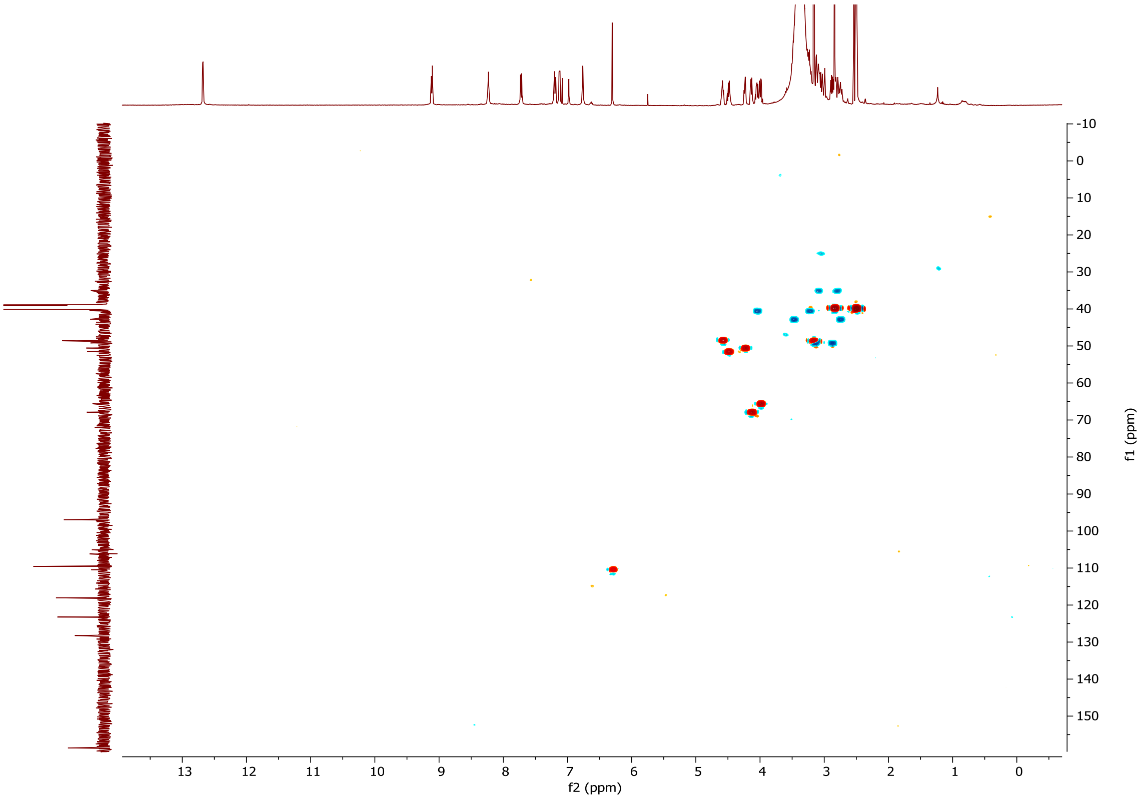

Figure S4 *g*-HSQC spectrum of Haloircinamide A (**1**) 500 MHz, DMSO

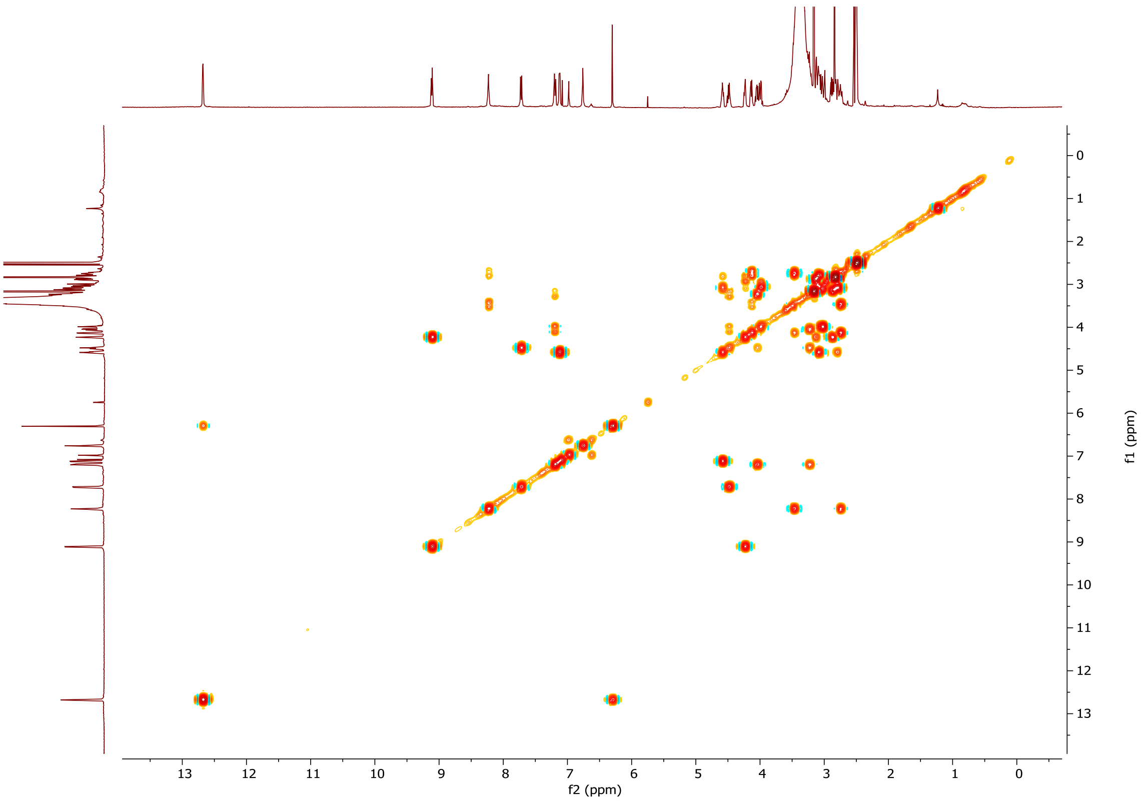

Figure S5 *g*-COSY spectrum of Haloircinamide A (**1**) 500 MHz, DMSO

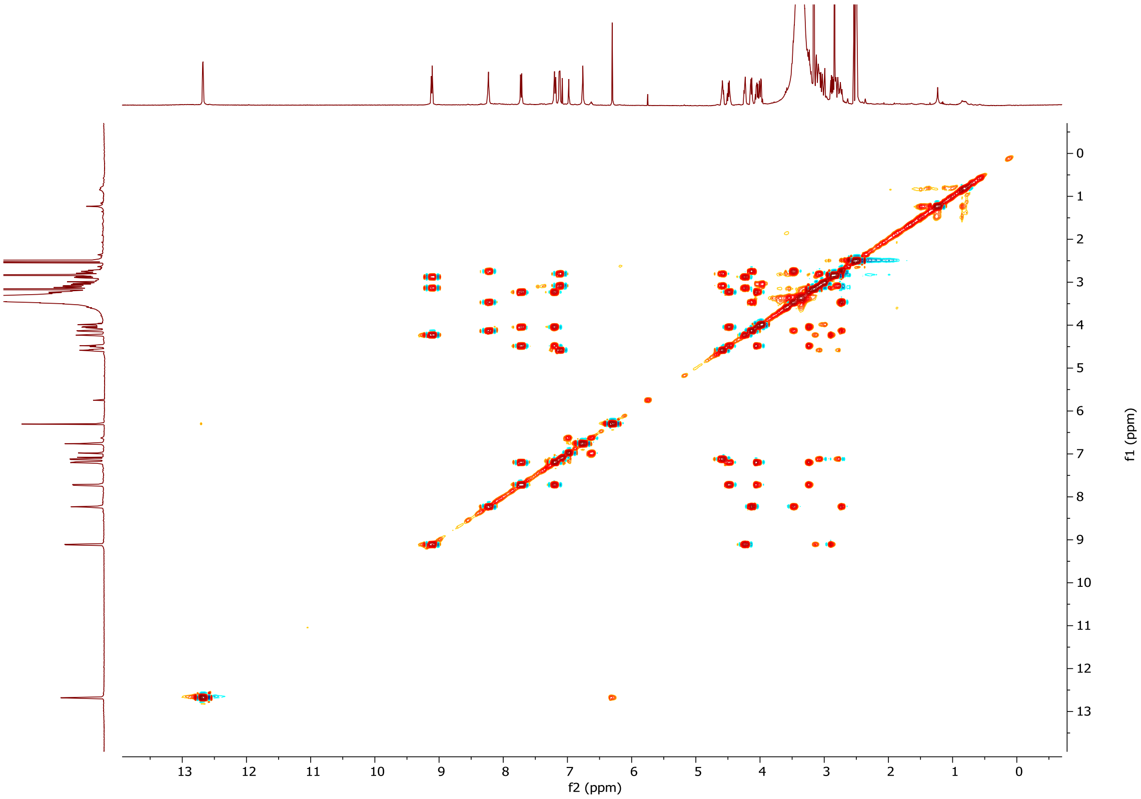

Figure S6 TOCSY spectrum of Haloircinamide A (**1**) 500 MHz, DMSO

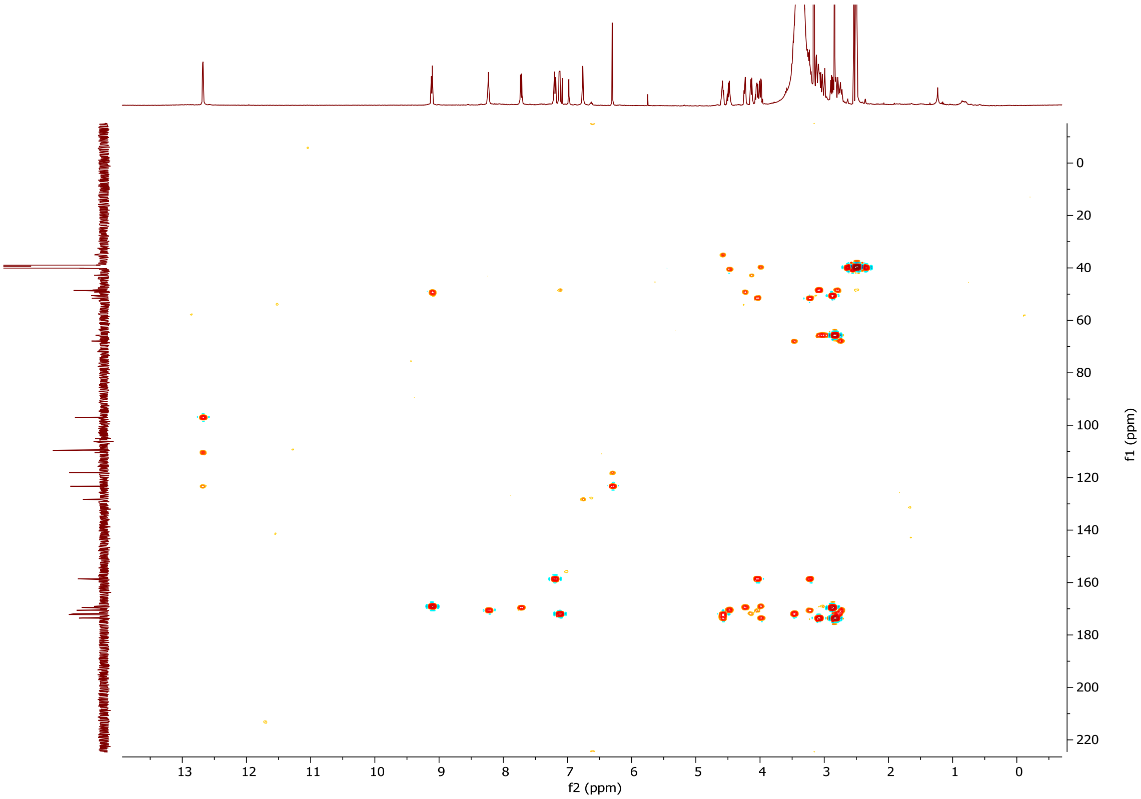

Figure S7 *g*-HMBC spectrum of Haloircinamide A (**1**) 500 MHz, DMSO

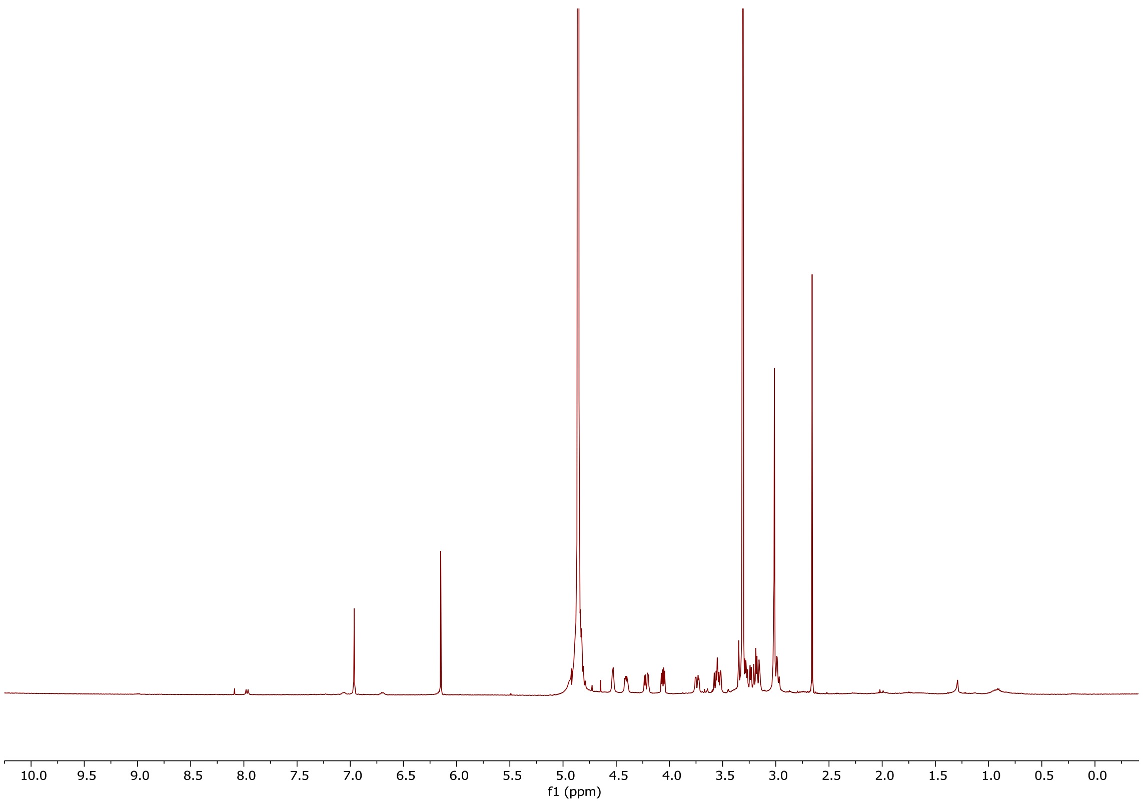

Figure S8 ^{1}H NMR spectrum of Haloircinamide A (**1**) 500 MHz, CD_3OD.

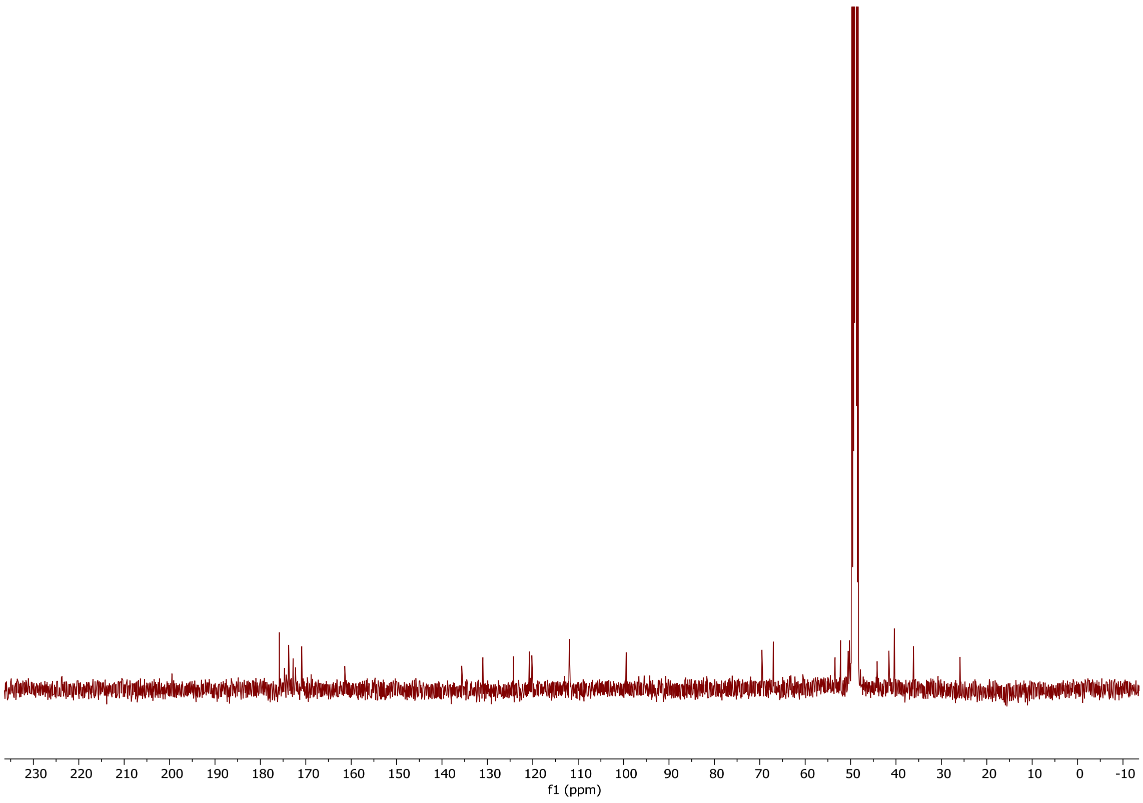

Figure S9 ^{13}C NMR spectrum of Haloircinamide A (**1**) 125 MHz, CD_3OD.

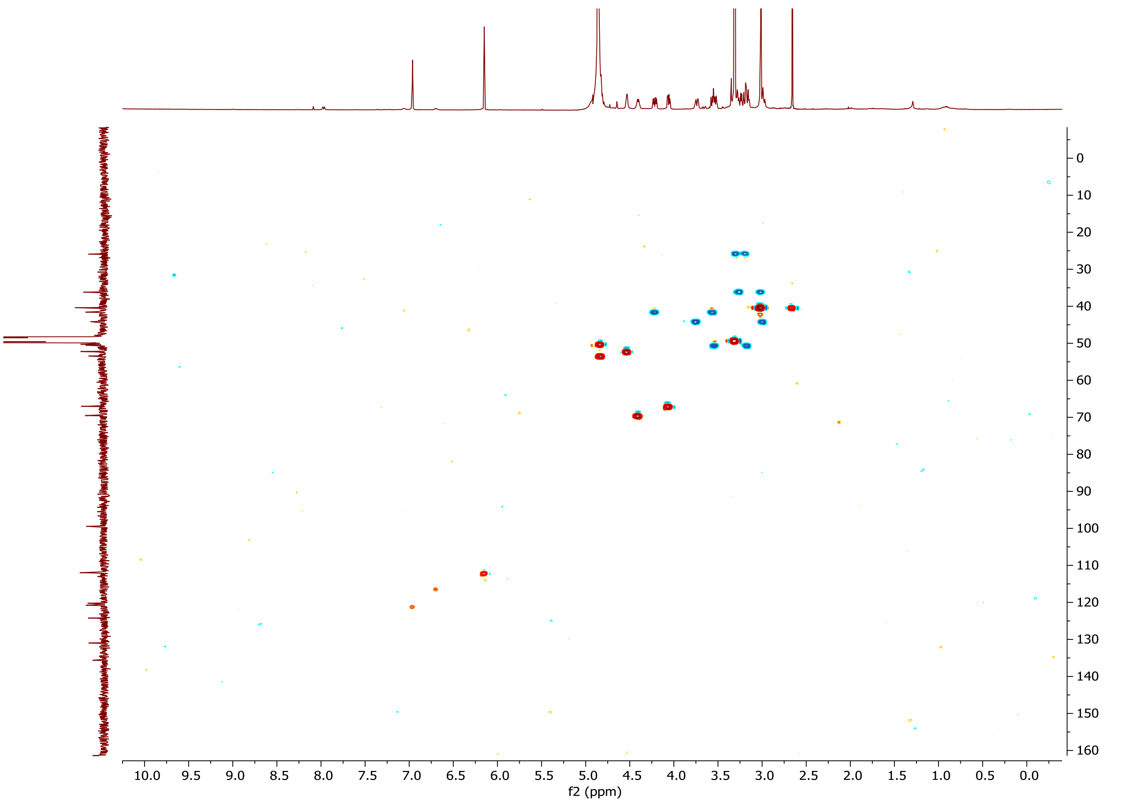

Figure S10 g-HSQC spectrum of Haloircinamide A (**1**) 500 MHz, CD_3OD

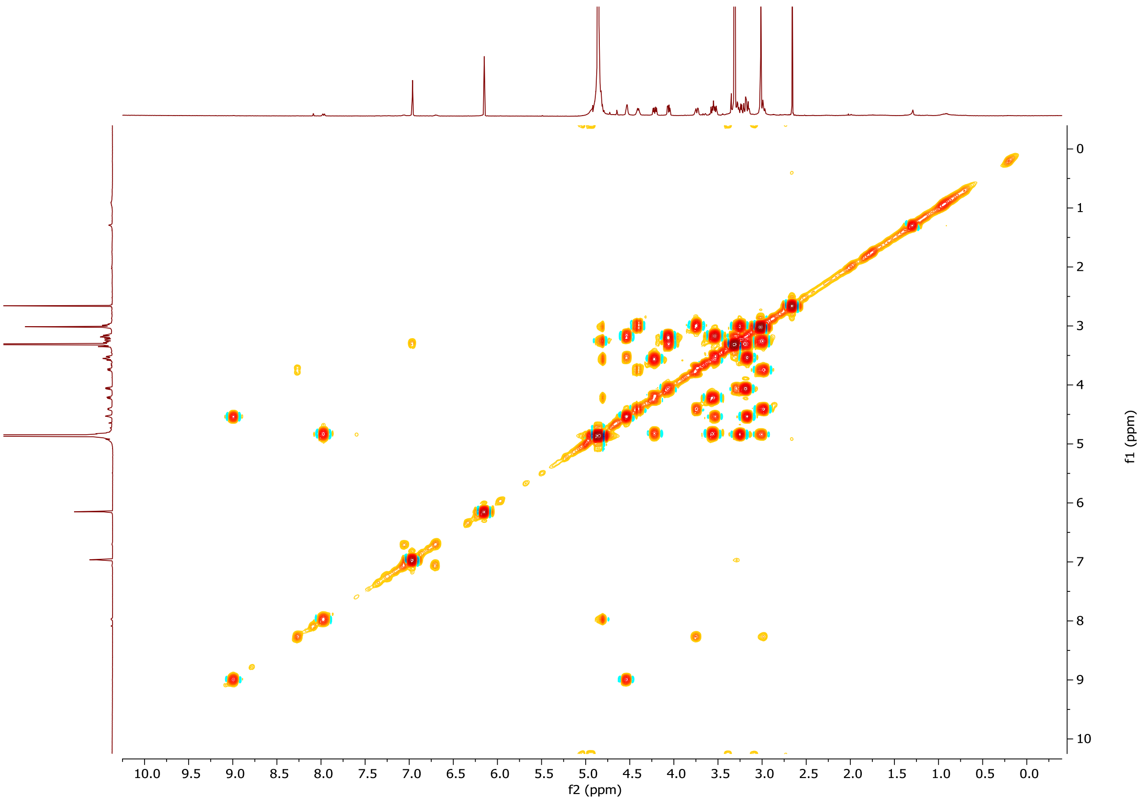

Figure S11 g-COSY spectrum of Haloircinamide A (**1**) 500 MHz, CD_3OD

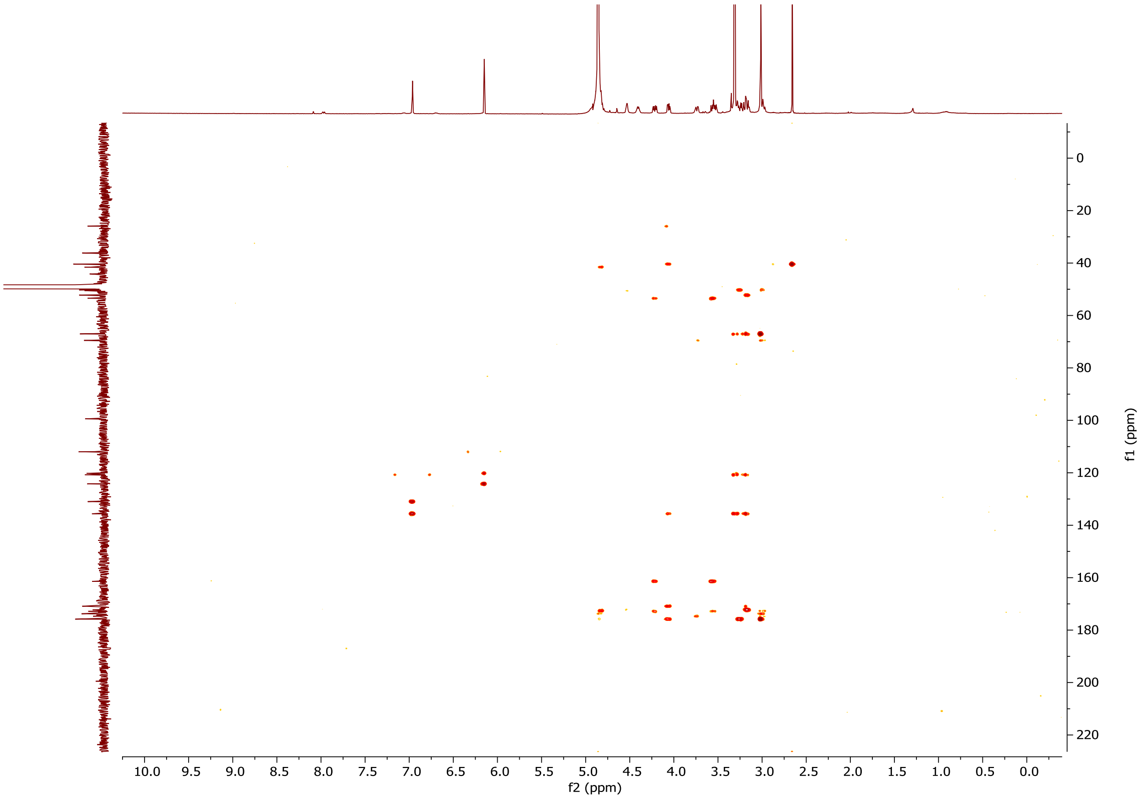

Figure S12 g-HMBC spectrum of Haloircinamide A (**1**) 500 MHz, CD_3OD

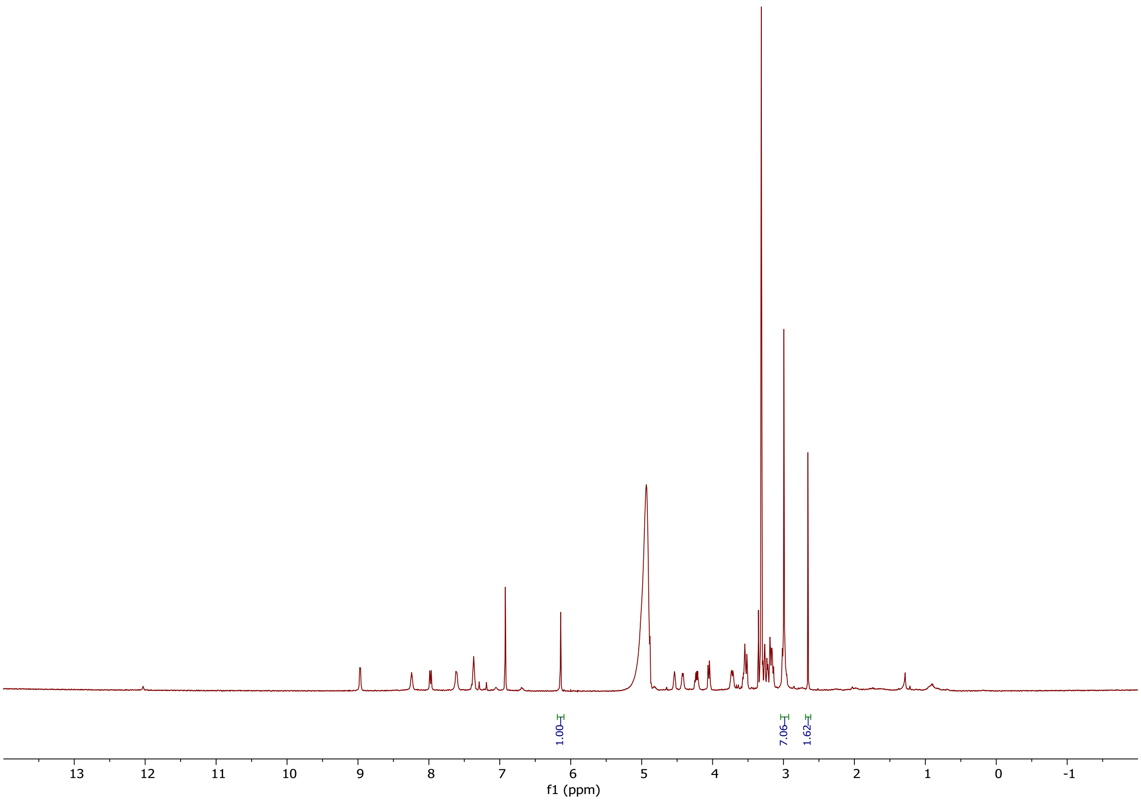

Figure S13 ^{1}H NMR spectrum of Haloircinamide A (**1**) 500 MHz, CD_3OH.

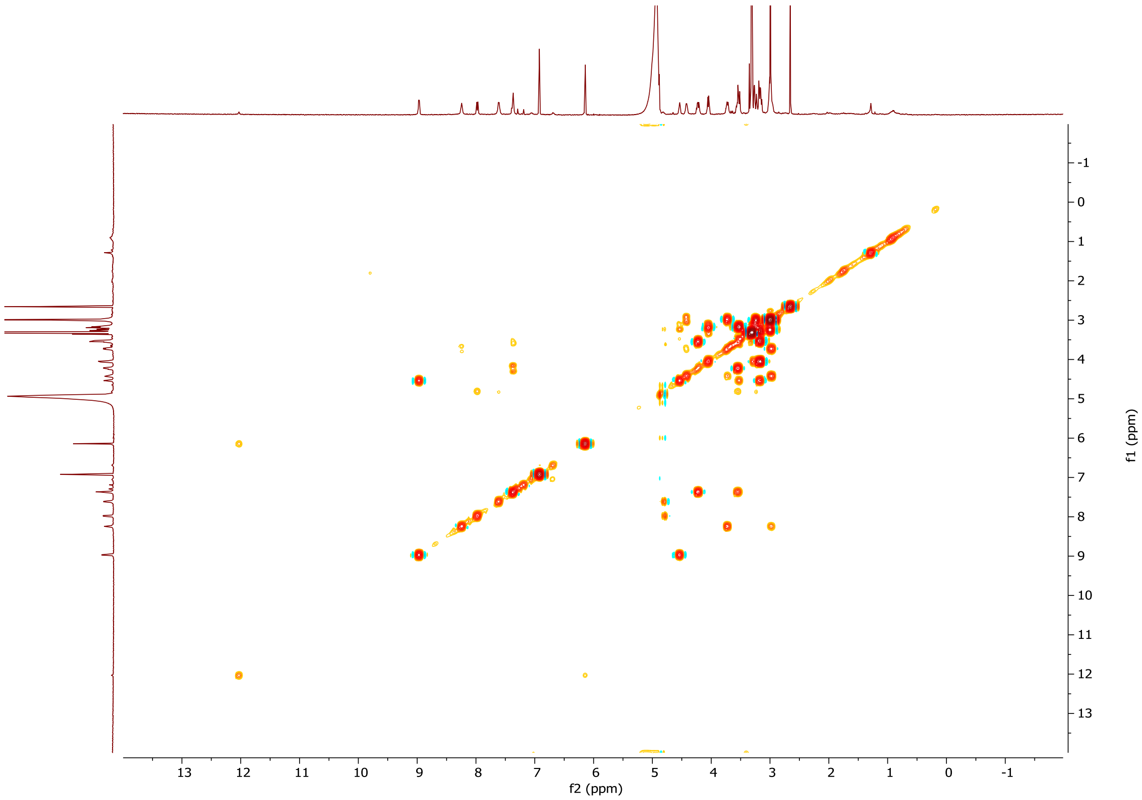

Figure S14 g-COSY spectrum of Haloircinamide A (**1**) 500 MHz, CD_3OH

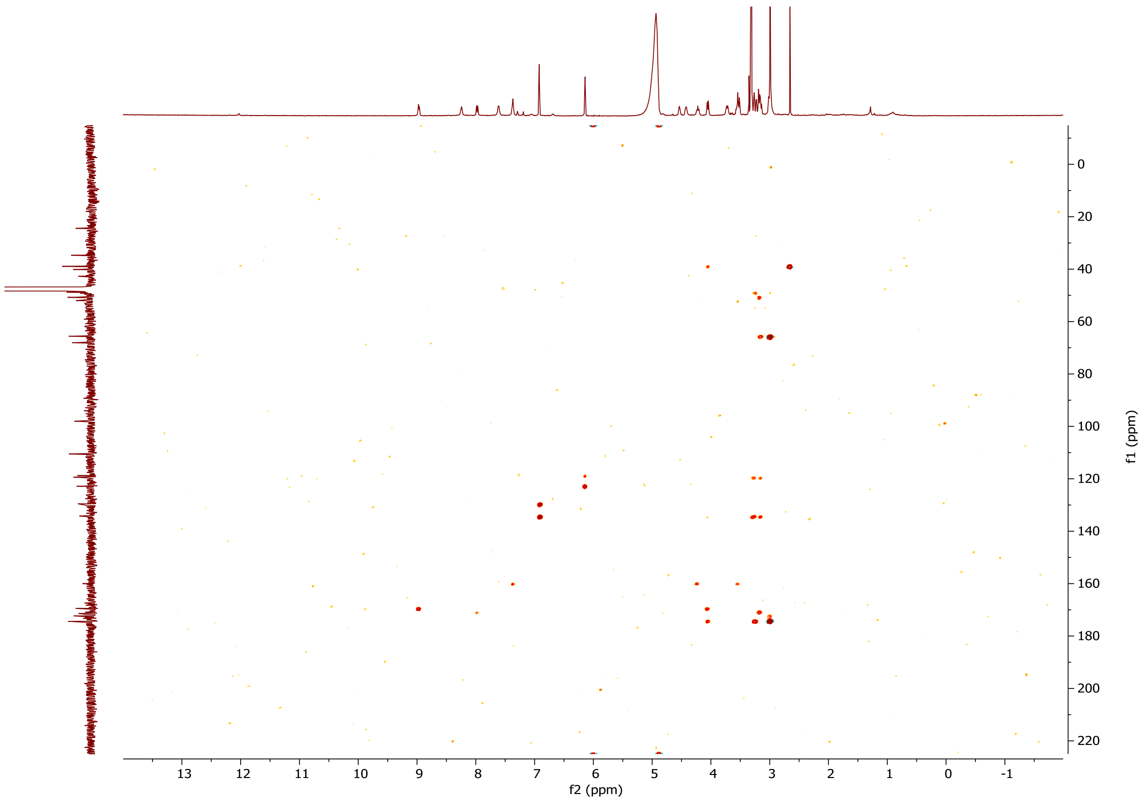

Figure S15 g-HMBC spectrum of Haloircinamide A (**1**) 500 MHz, CD_3OH

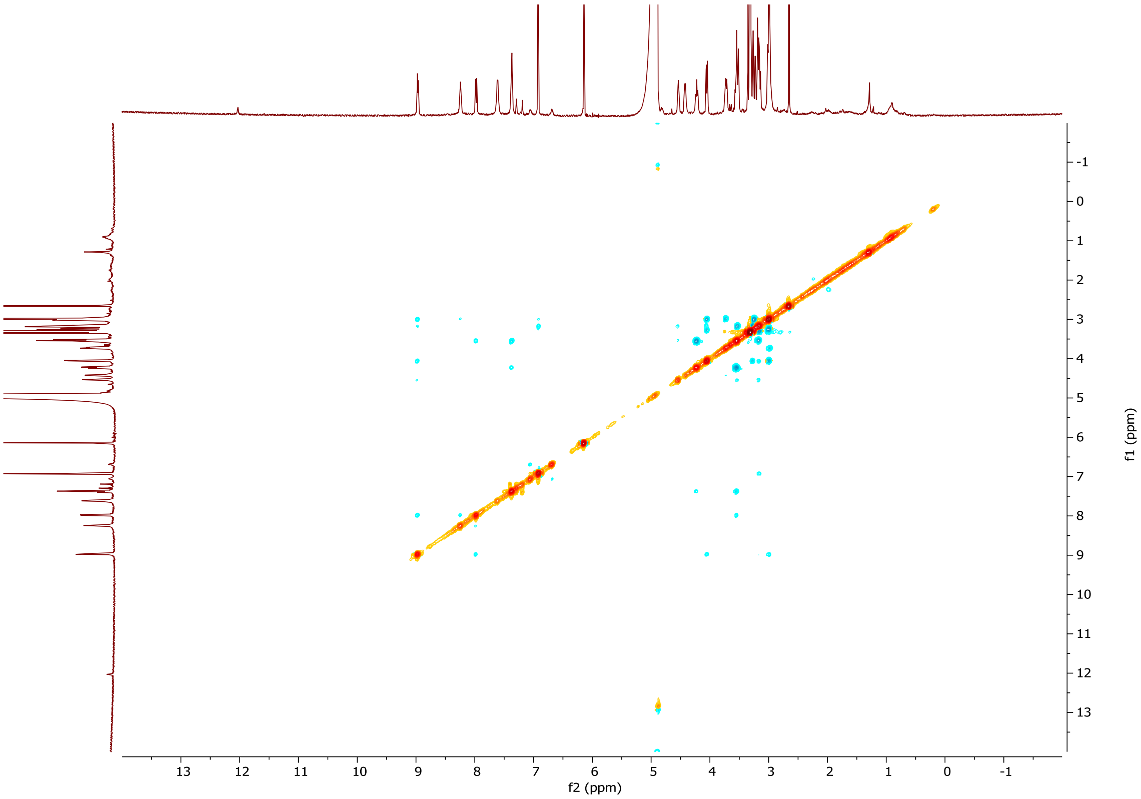

Figure S16 ROESY spectrum of Haloircinamide A (**1**) 500 MHz, CD_3OH

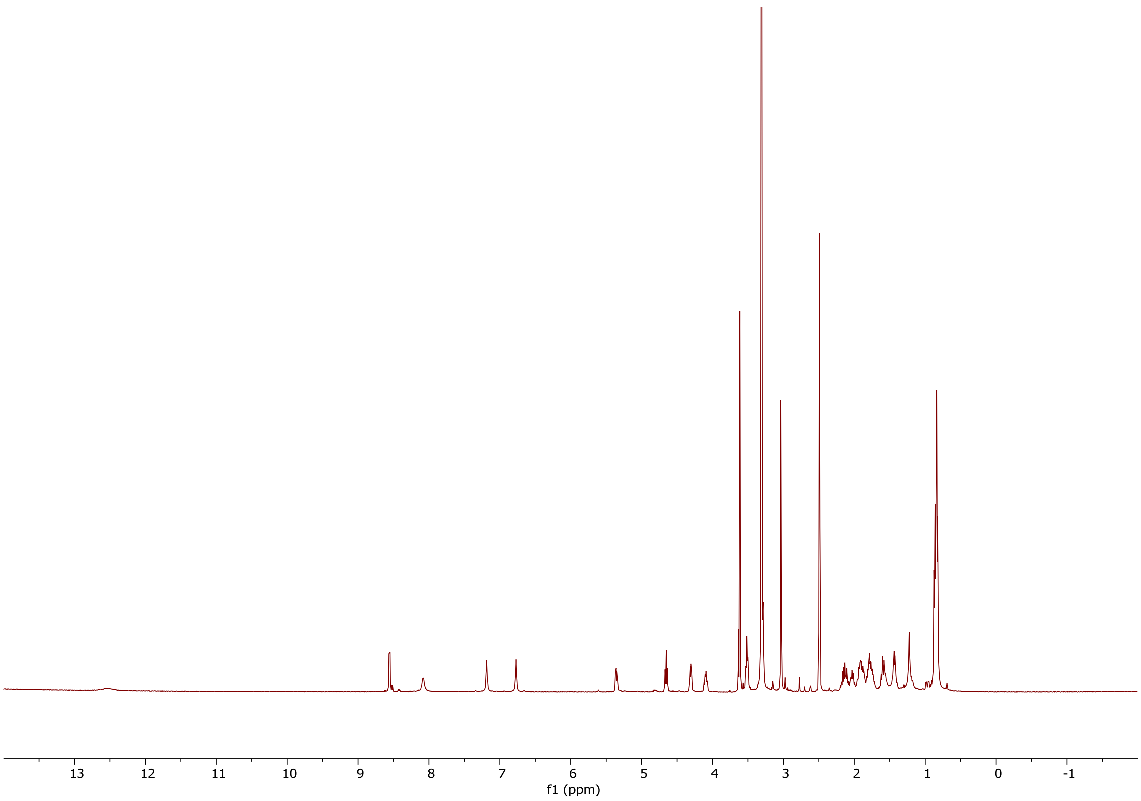

Figure S17 ^{1}H NMR spectrum of Seribunamide A (**2**), 500 MHz, DMSO

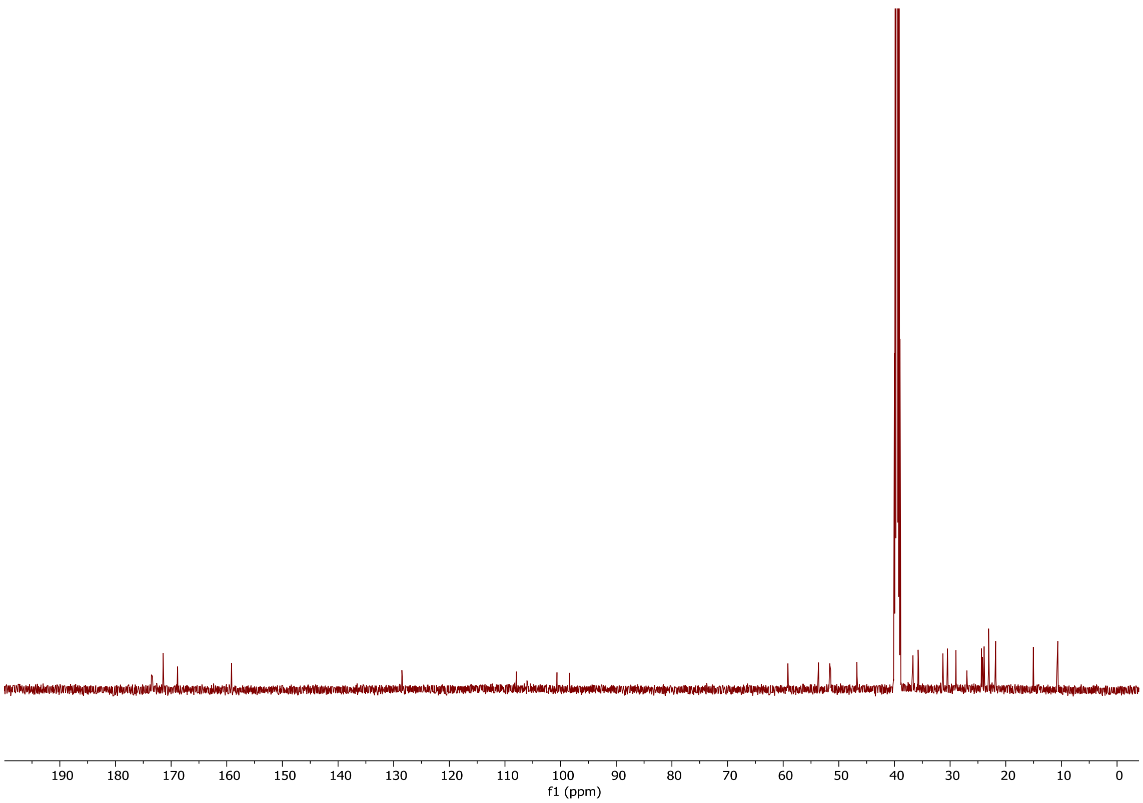

Figure S18 ^{13}C NMR spectrum of Seribunamide A (**2**), 125 MHz, DMSO

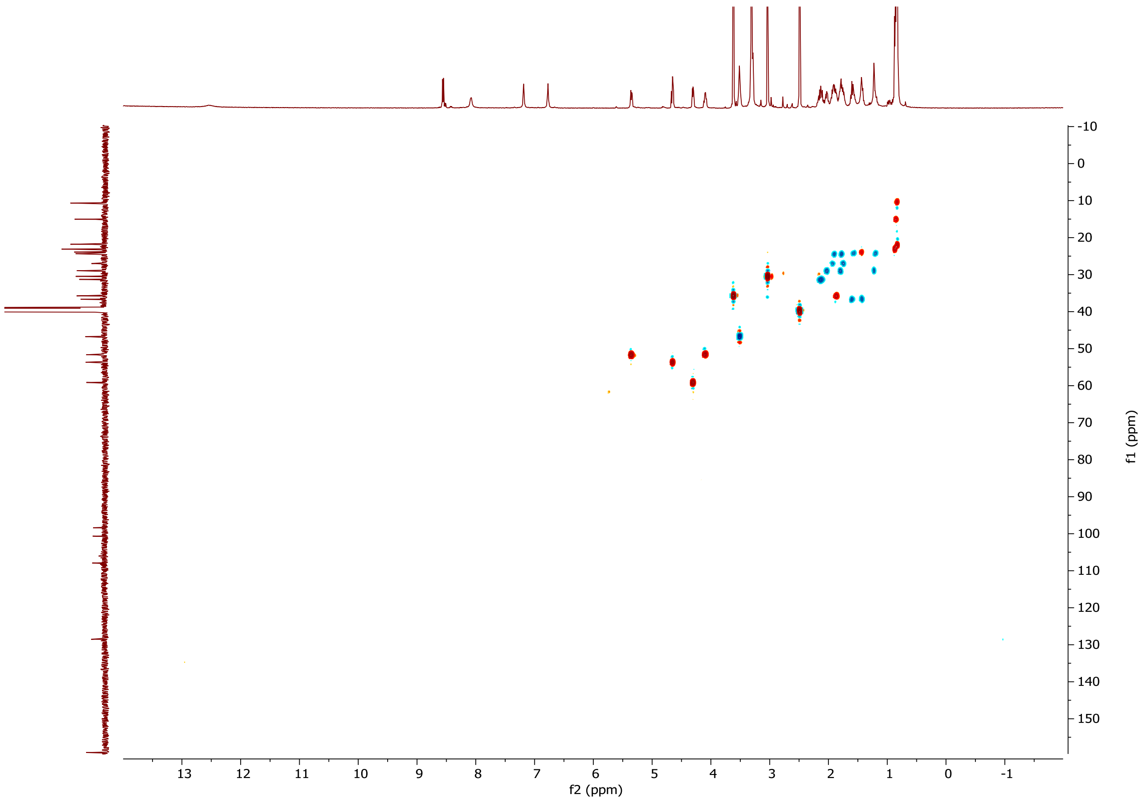

Figure S19 gHSQC spectrum of Seribunamide A (**2**), 500 MHz, DMSO

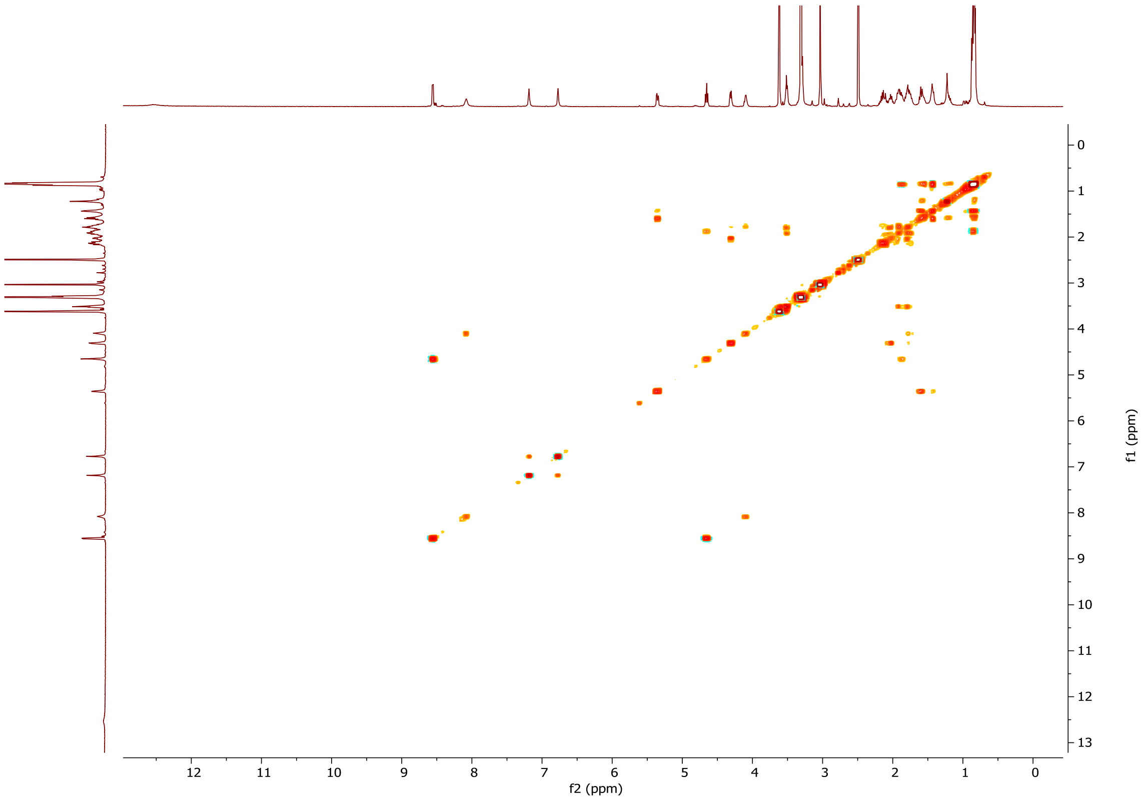

Figure S20 gCOSY spectrum of Seribunamide A (**2**), 500 MHz, DMSO

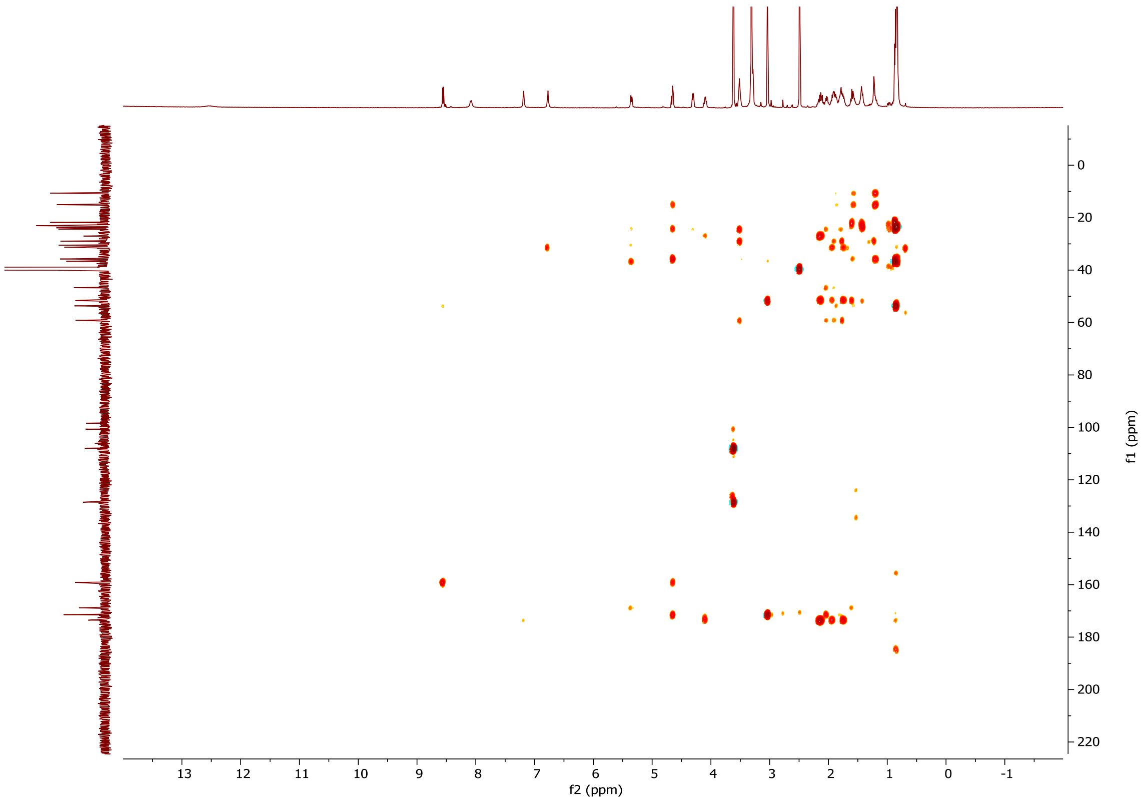

Figure S21 gHMBC spectrum of Seribunamide A (**2**), 500 MHz, DMSO

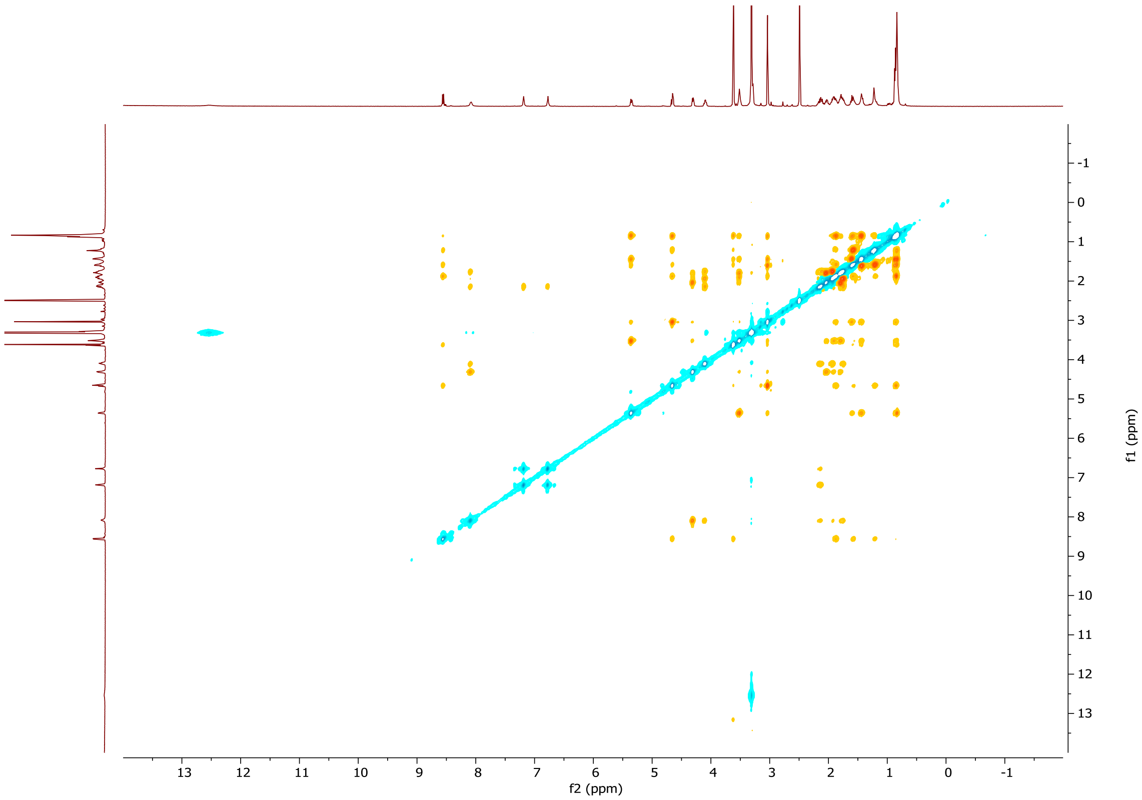

Figure S22 ROESY spectrum of Seribunamide A (**2**), 500 MHz, DMSO

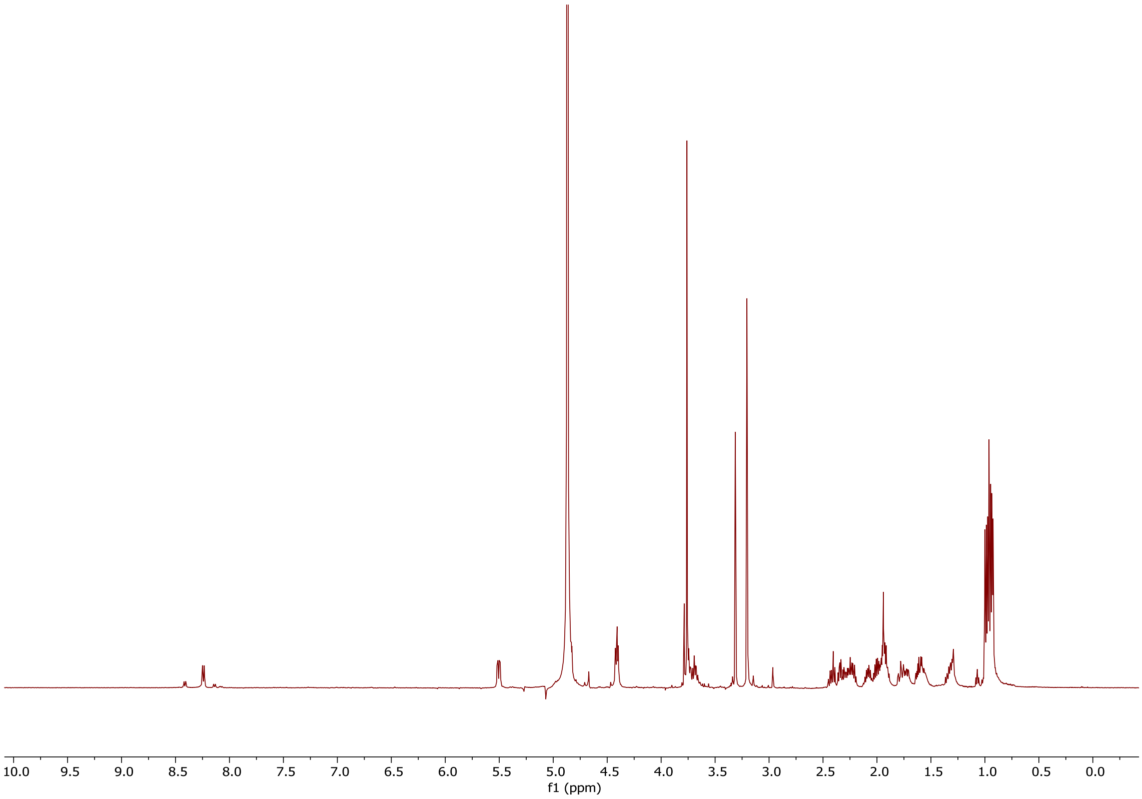

Figure S23 ^{1}H NMR spectrum of Seribunamide A (**2**), 500 MHz, CD_3OD

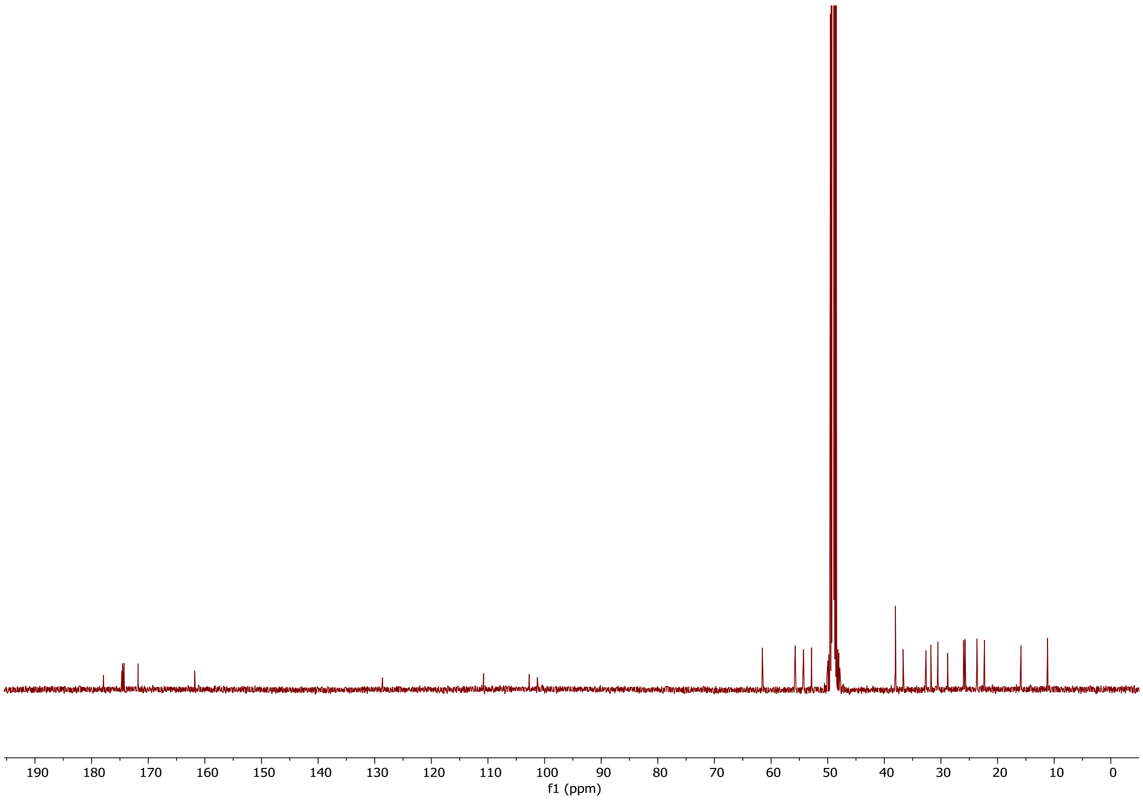

Figure S24 ^{13}C NMR spectrum of Seribunamide A (**2**), 125 MHz, CD_3OD

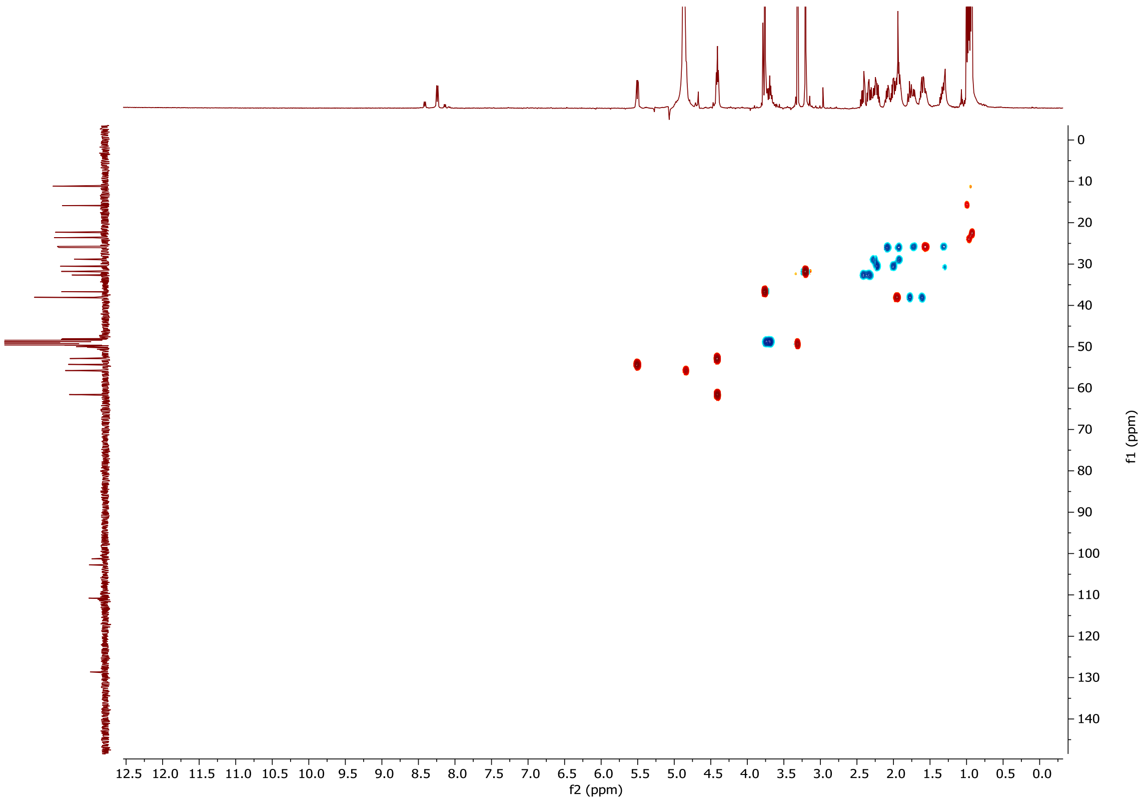

Figure S25 gHSQC spectrum of Seribunamide A (**2**), 500 MHz, CD_3OD

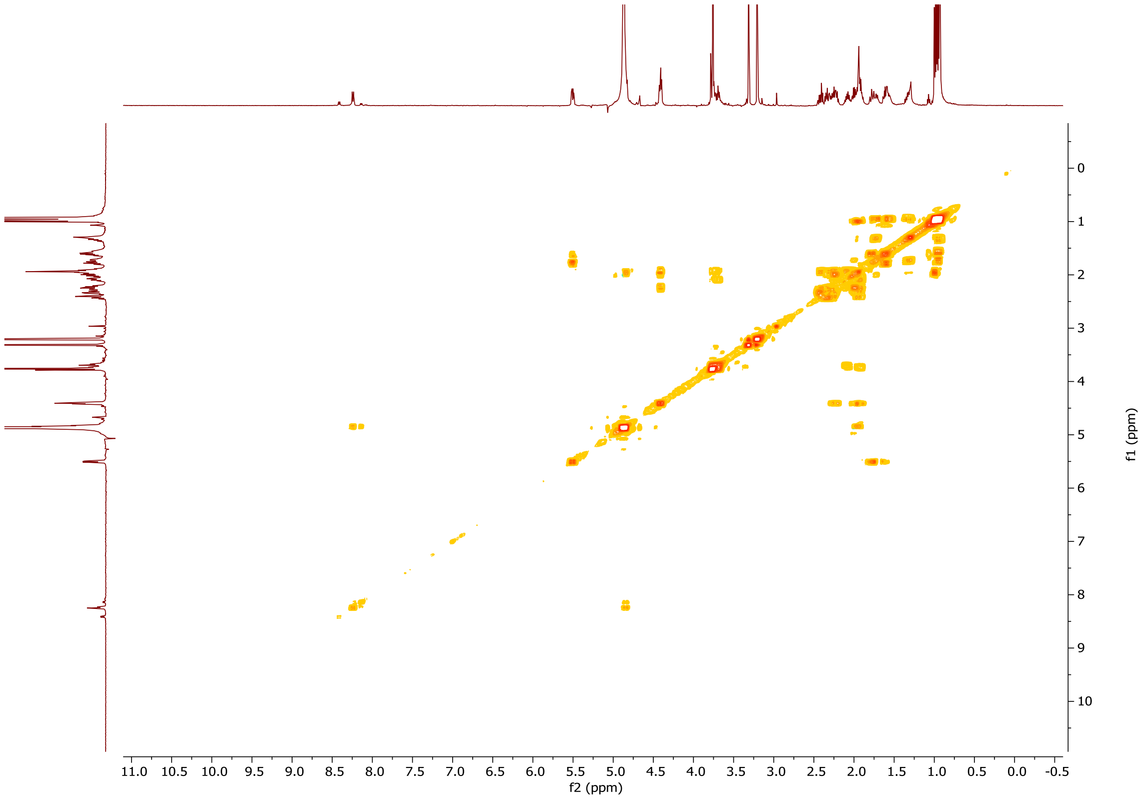

Figure S26 gCOSY spectrum of Seribunamide A (**2**), 500 MHz, CD_3OD

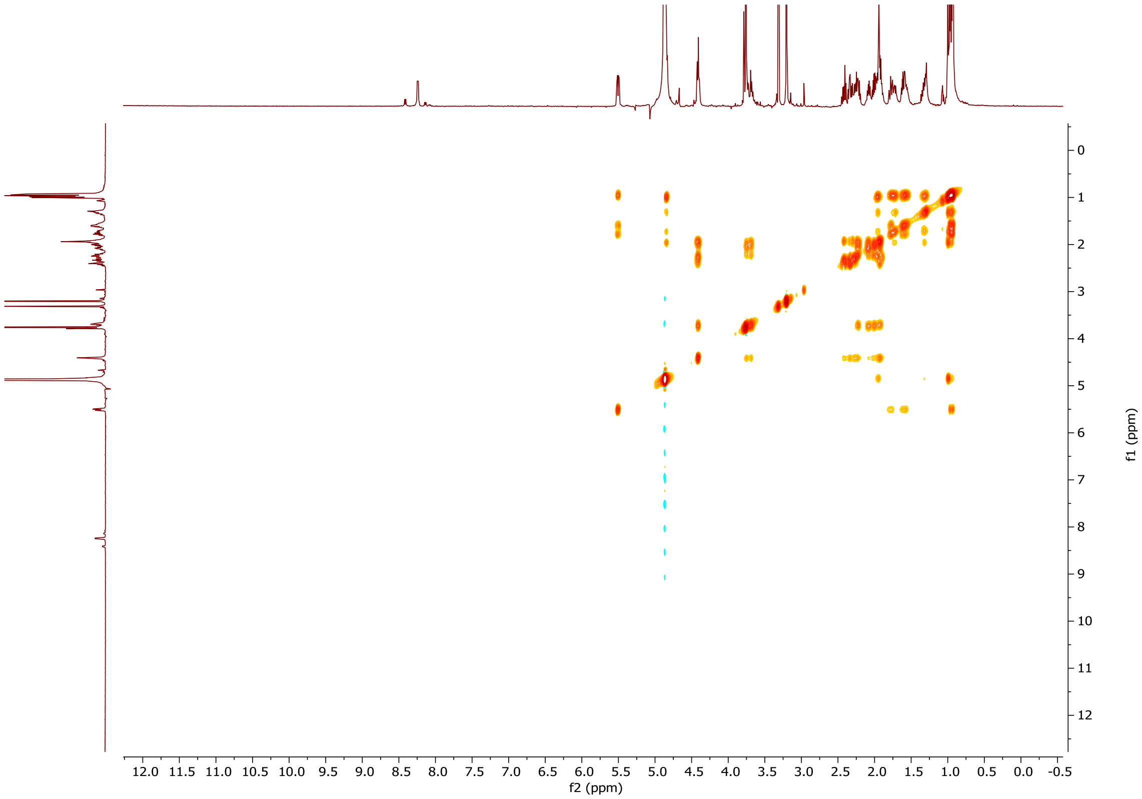

Figure S27 TOCSY spectrum of Seribunamide A (**2**), 500 MHz, CD_3OD

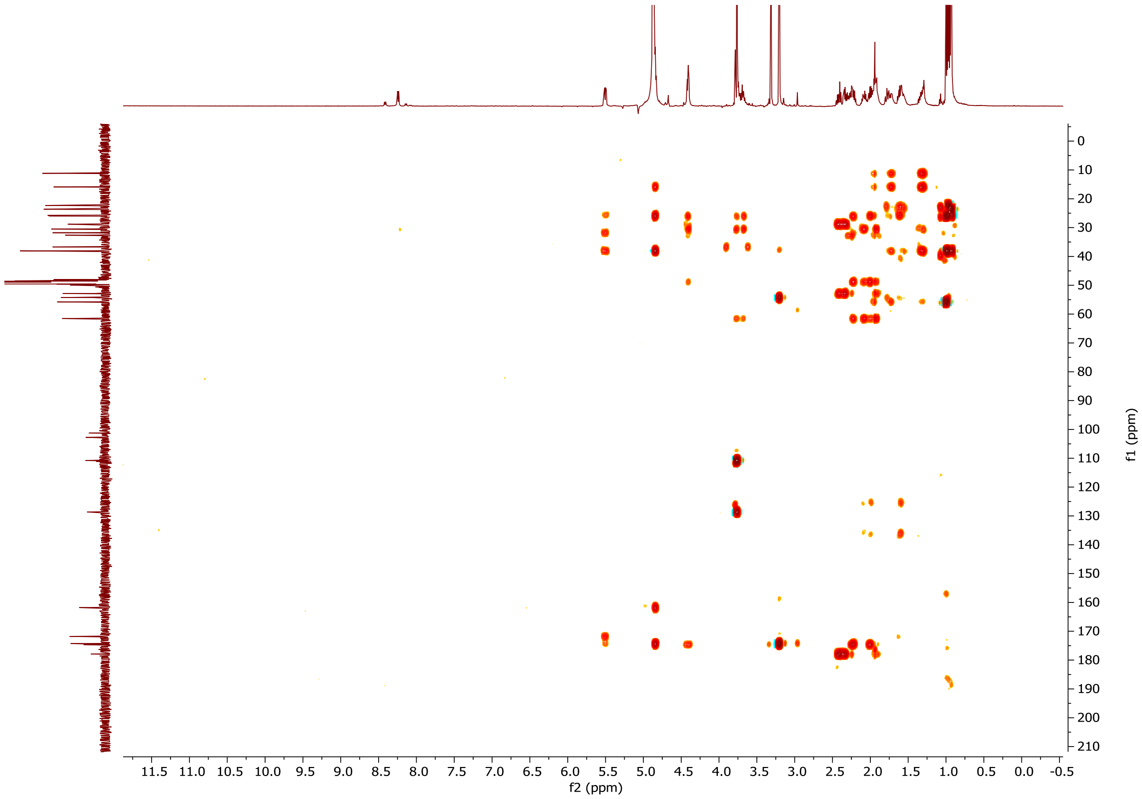

Figure S28 gHMBC spectrum of Seribunamide A (**2**), 500 MHz, CD_3OD

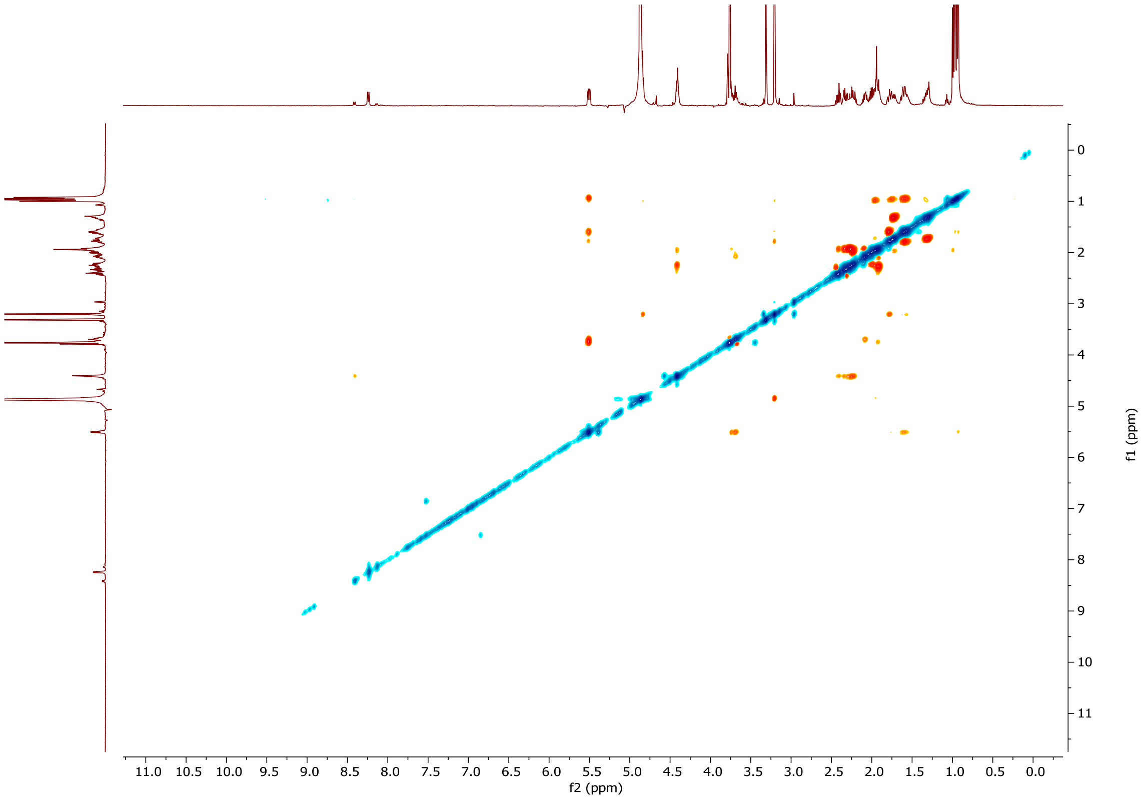

Figure S29 ROESY spectrum of Seribunamide A (**2**), 500 MHz, CD_3OD

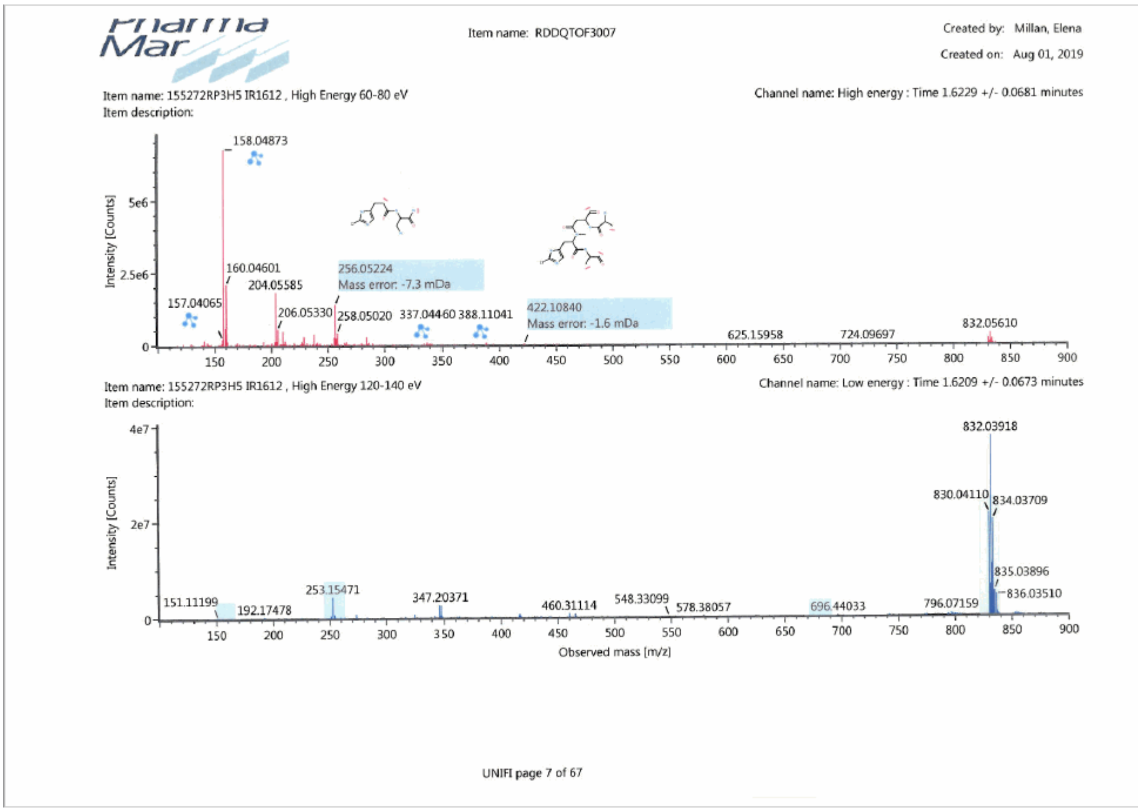

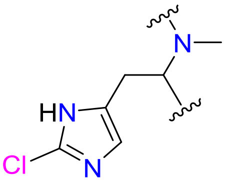

Observed *m/z* 158.0487
Expected *m/z* 158.0480
Mass error 4.92 ppm

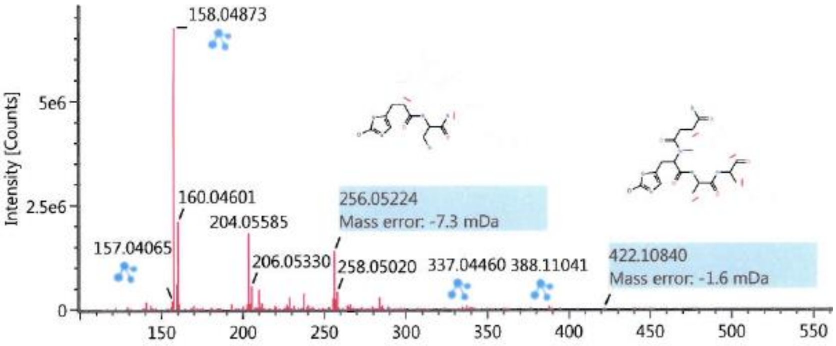

Figure S30 QTOFMS spectrum of Haloirciamide A (**1**) and fragments found

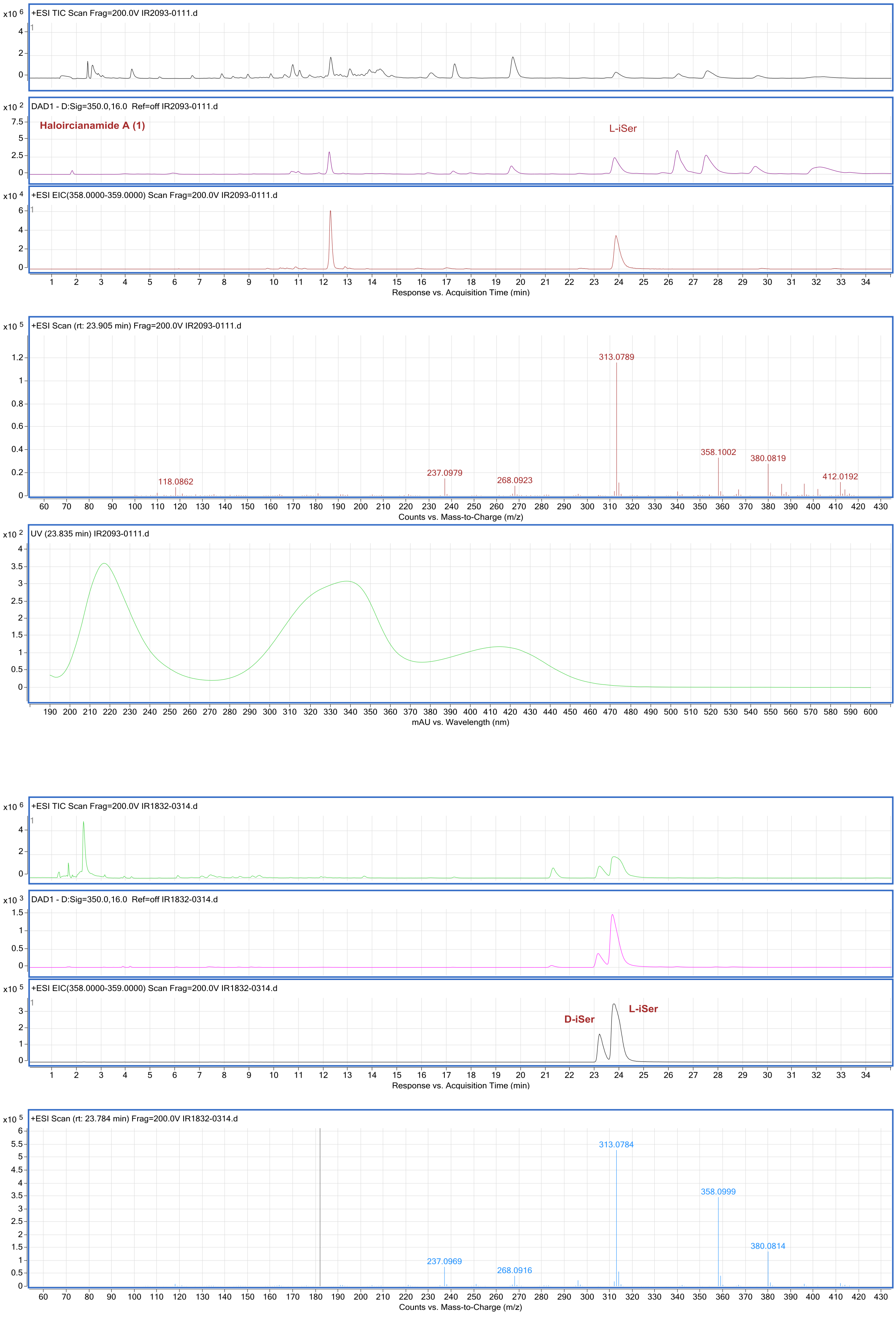

Figure S31 Marfey of Haloirciamide A (**1**) and iSer standards

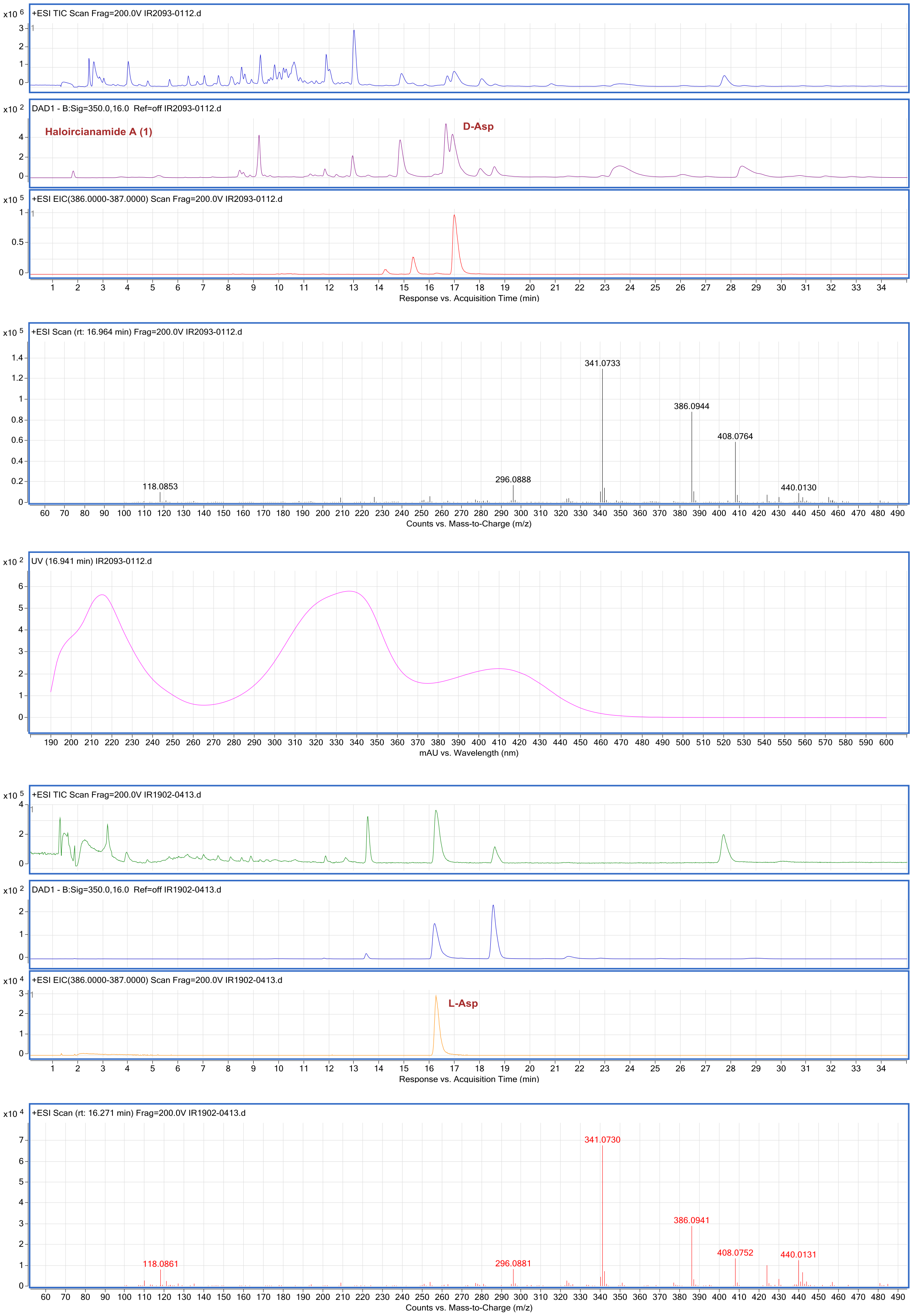

+ESI TIC Scan Frag=200.0V IR2093-0112.d
DAD1 - B:Sig=350.0,16.0 Ref=off IR2093-0112.d
Haloircianamide A (1)
D-Asp
+ESI EIC(386.0000-387.0000) Scan Frag=200.0V IR2093-0112.d
Response vs. Acquisition Time (min)
+ESI Scan (rt: 16.964 min) Frag=200.0V IR2093-0112.d
341.0733
386.0944
408.0764
296.0888
118.0853
440.0130
Counts vs. Mass-to-Charge (m/z)
UV (16.941 min) IR2093-0112.d
mAU vs. Wavelength (nm)
+ESI TIC Scan Frag=200.0V IR1902-0413.d
DAD1 - B:Sig=350.0,16.0 Ref=off IR1902-0413.d
+ESI EIC(386.0000-387.0000) Scan Frag=200.0V IR1902-0413.d
L-Asp
Response vs. Acquisition Time (min)
+ESI Scan (rt: 16.271 min) Frag=200.0V IR1902-0413.d
341.0730
386.0941
408.0752
440.0131
118.0861
296.0881
Counts vs. Mass-to-Charge (m/z)

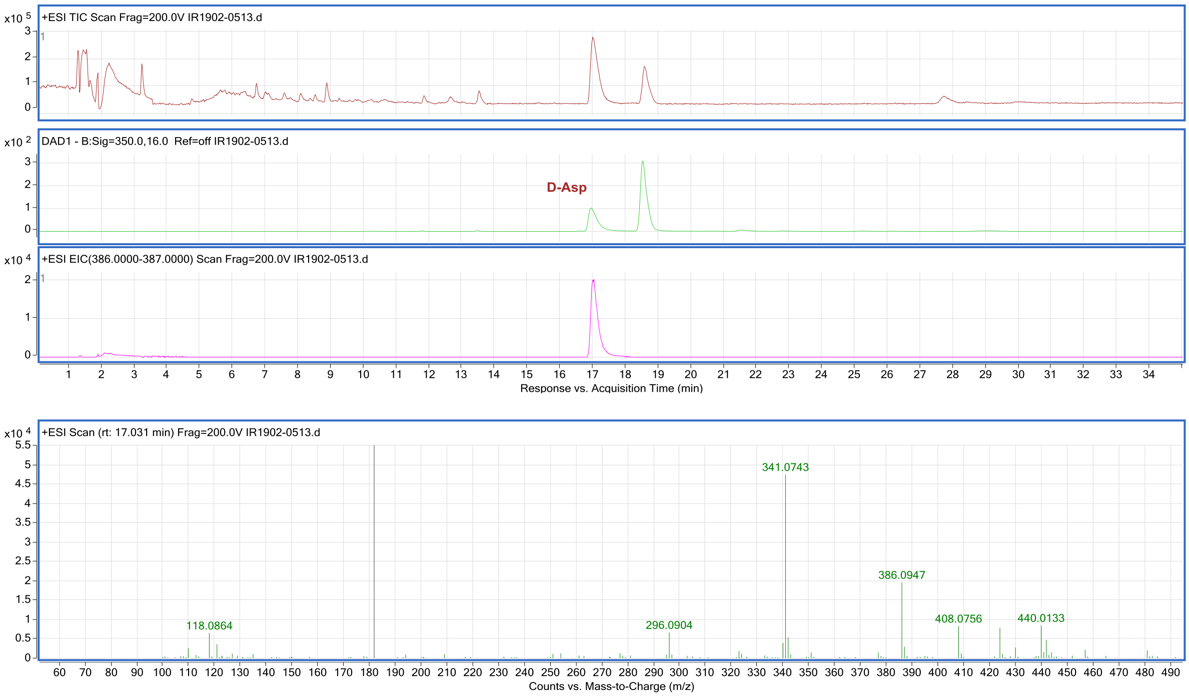

Figure S32 Marfey of Haloirciamide A (**1**) and Asp standards

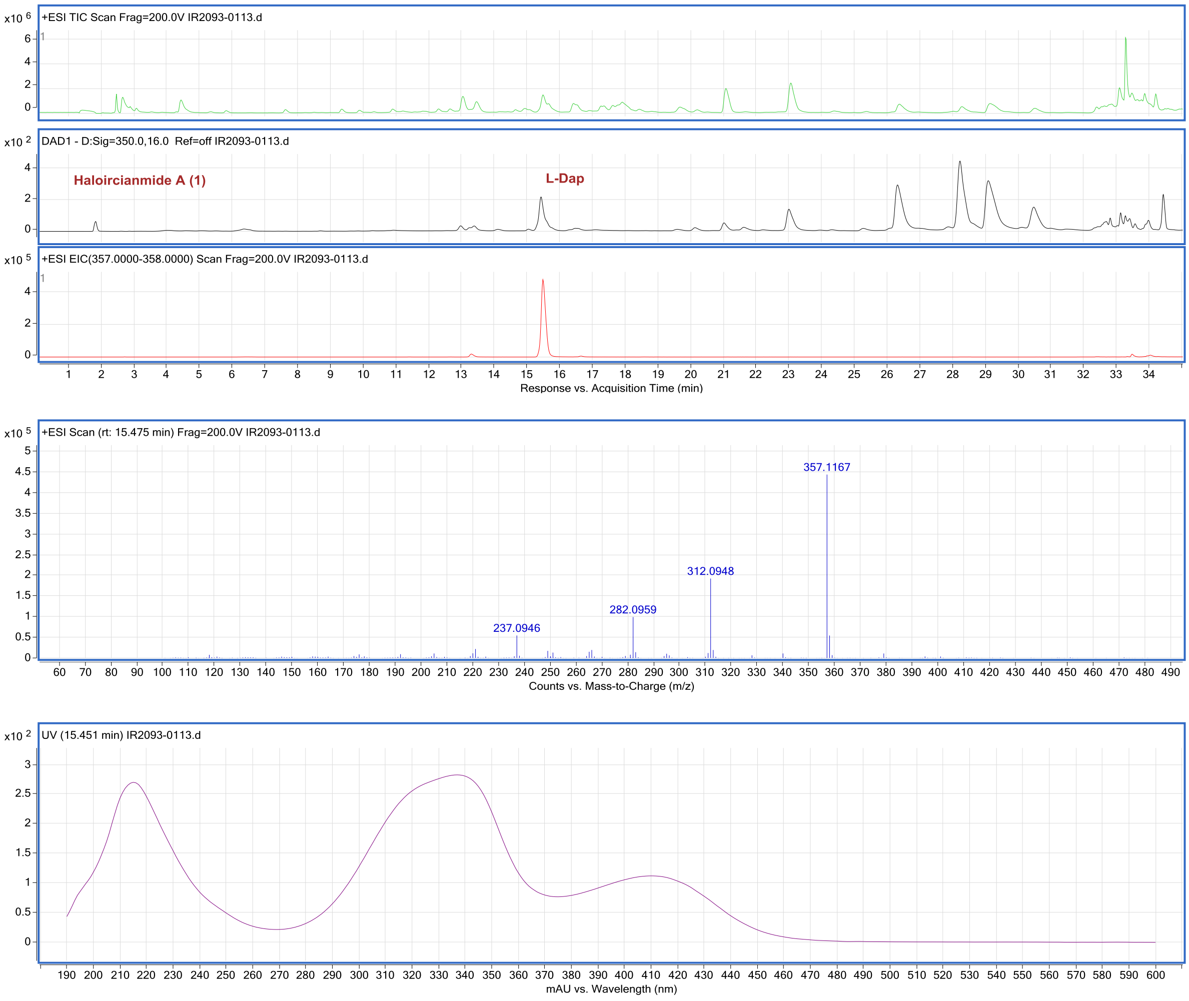

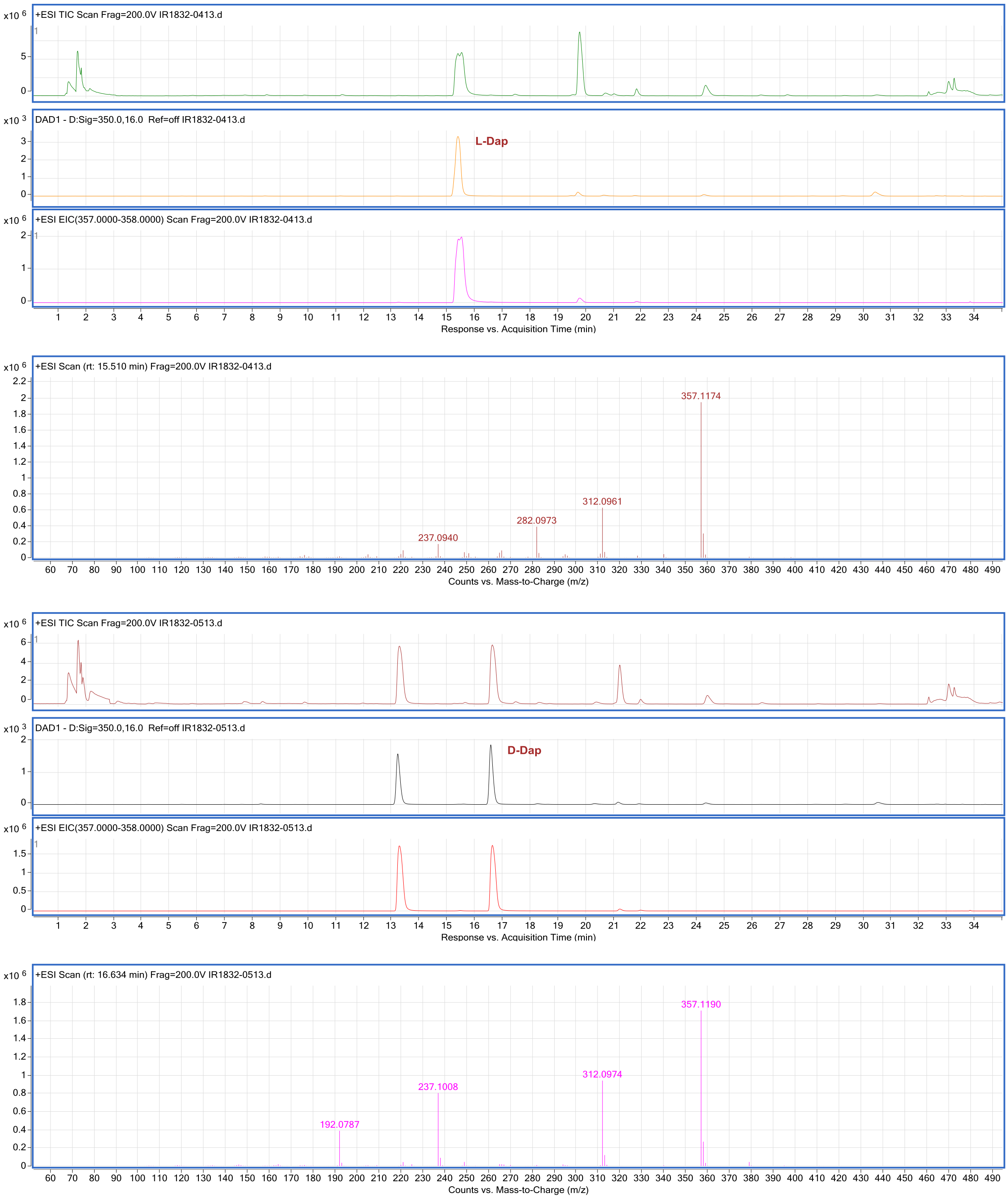

Figure S33 Marfey of Haloirciamide A (**1**) and Dap standards

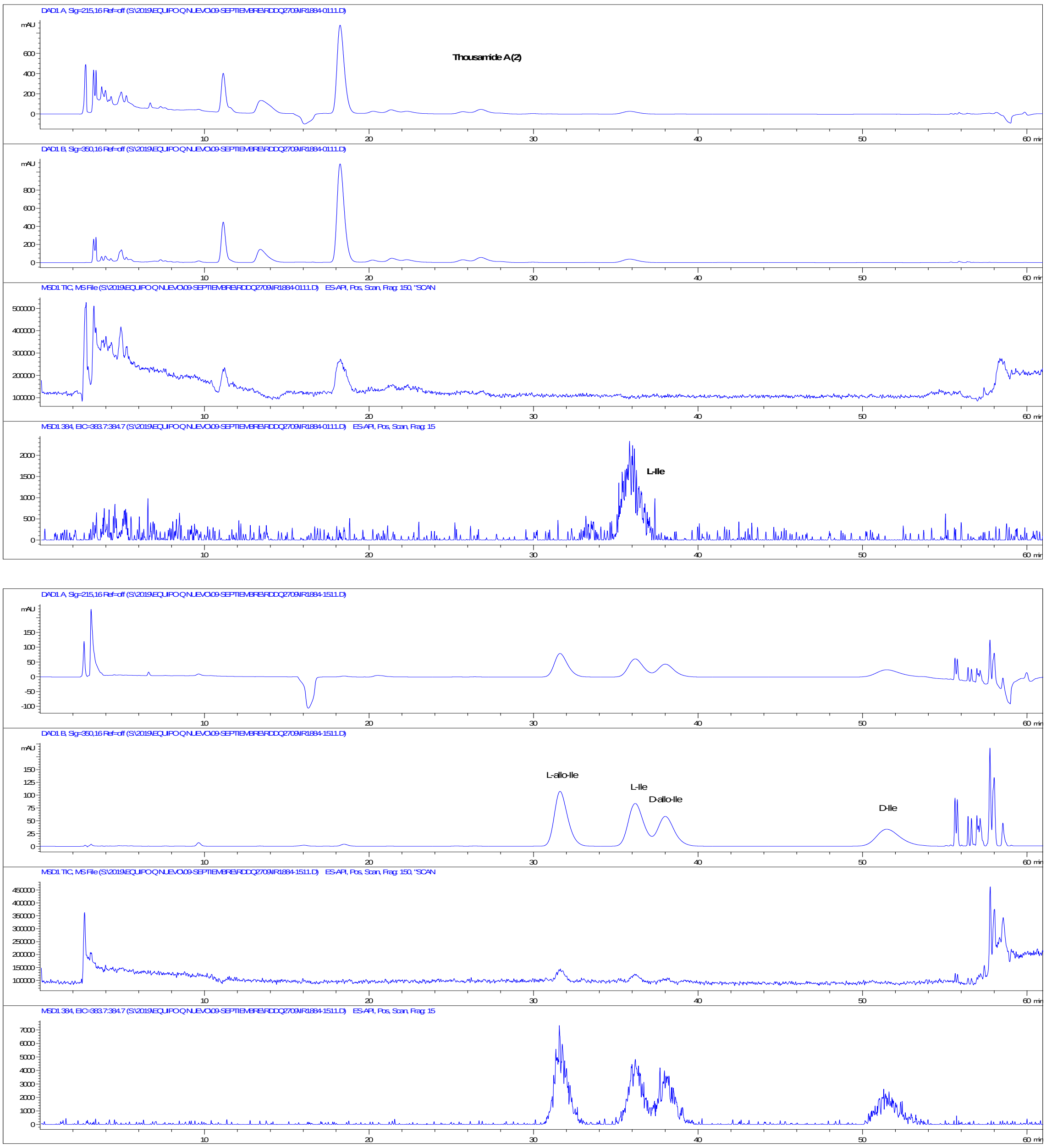

Figure S34 Marfey of Seribunamide A (**2**) and Ile standards

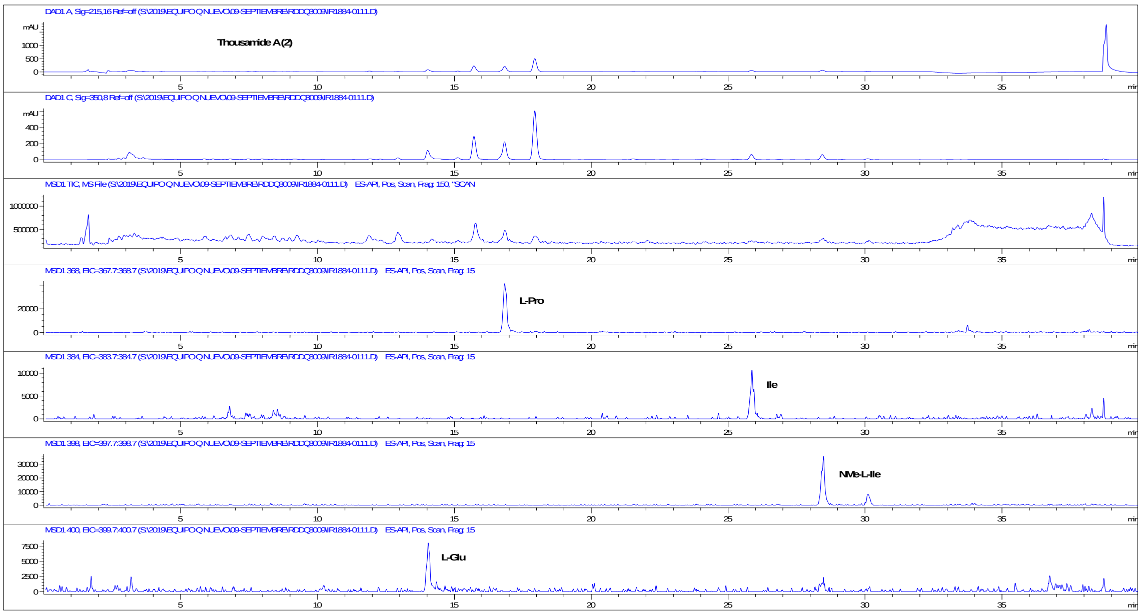

Figure S35 Marfey of Seribunamide A (**2**)

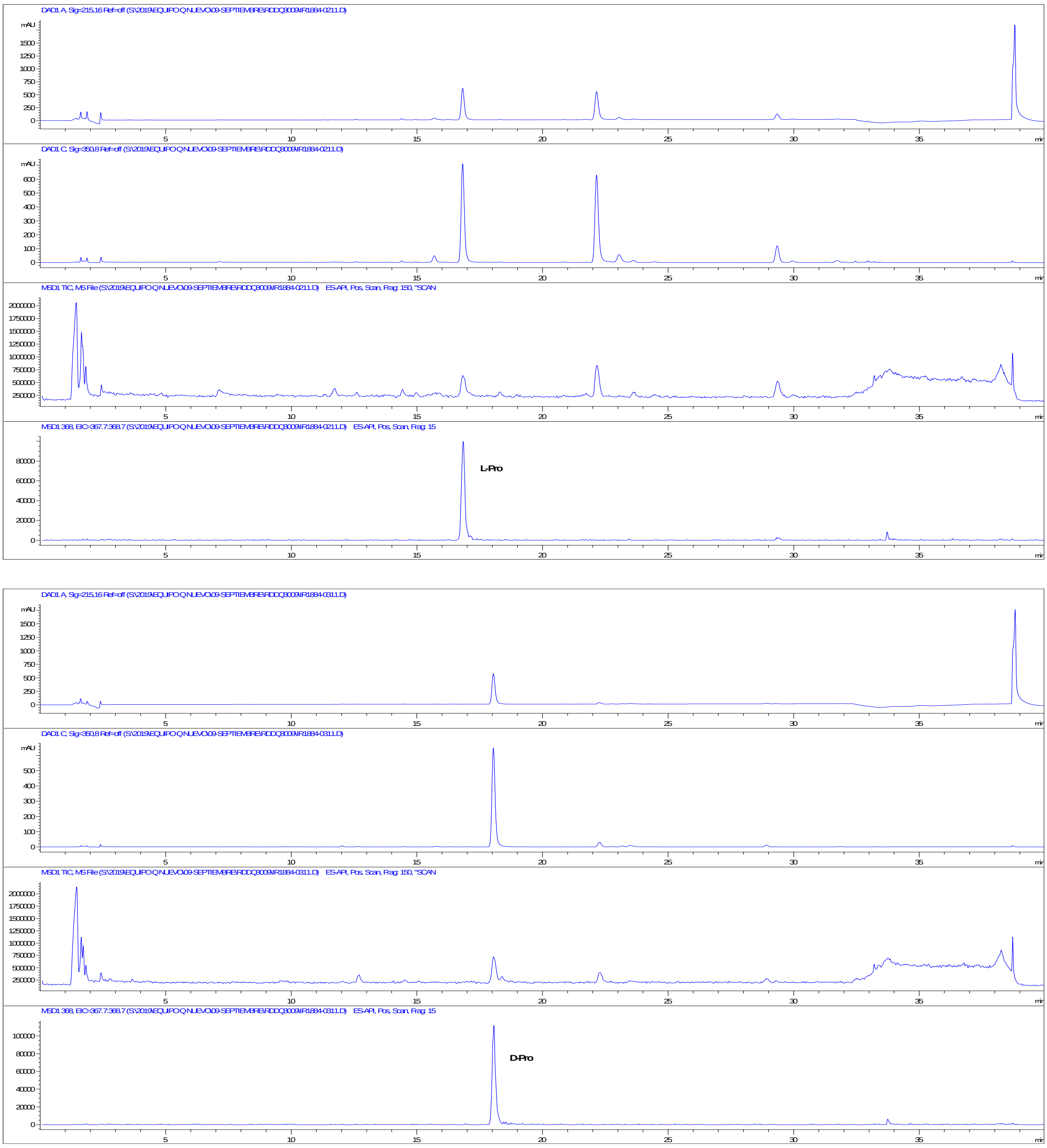

Figure S36 Marfey of Seribunamide A (**2**). Pro standards

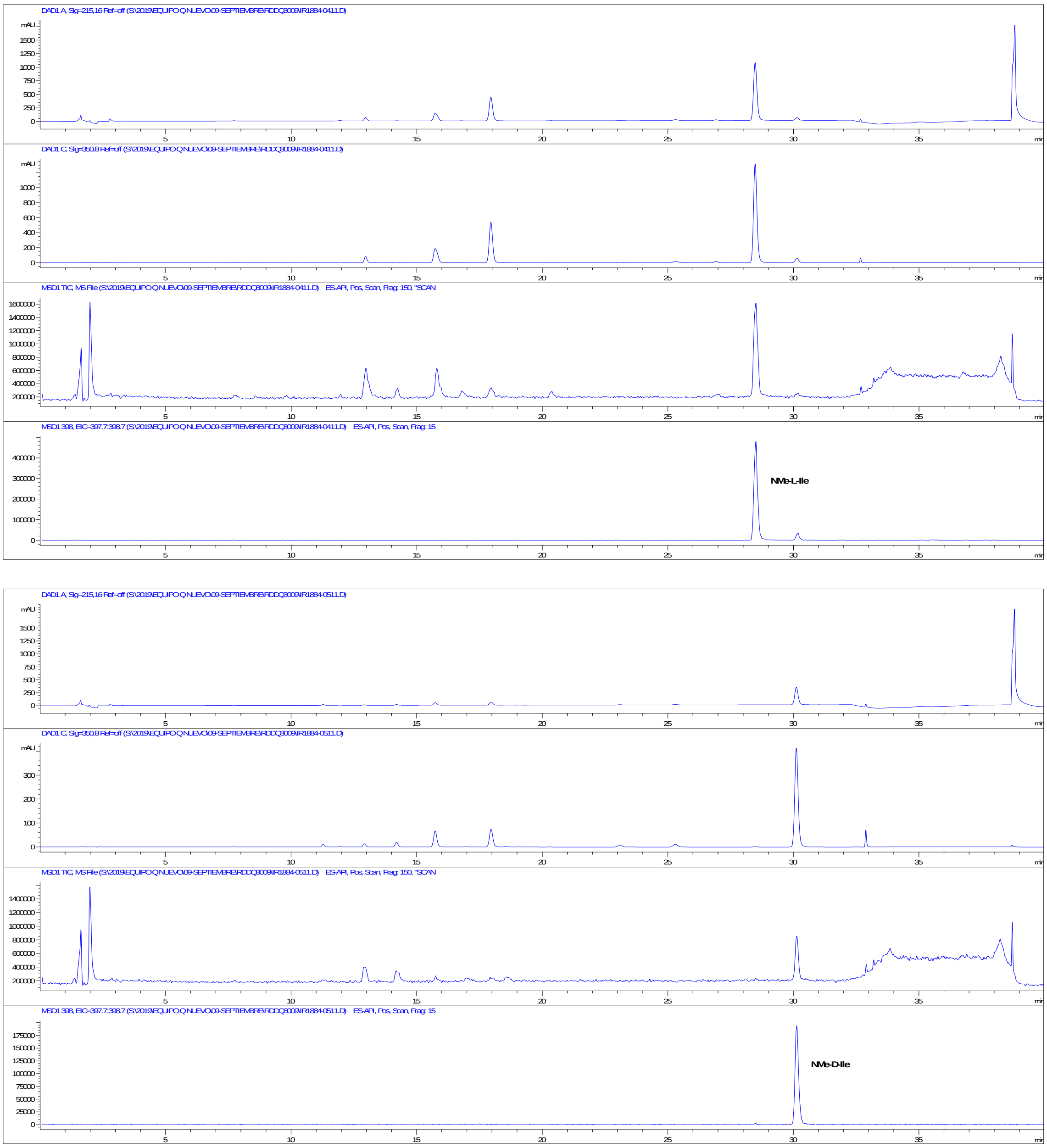

Figure S37 Marfey of Seribunamide A (**2**). NMeLeu standards

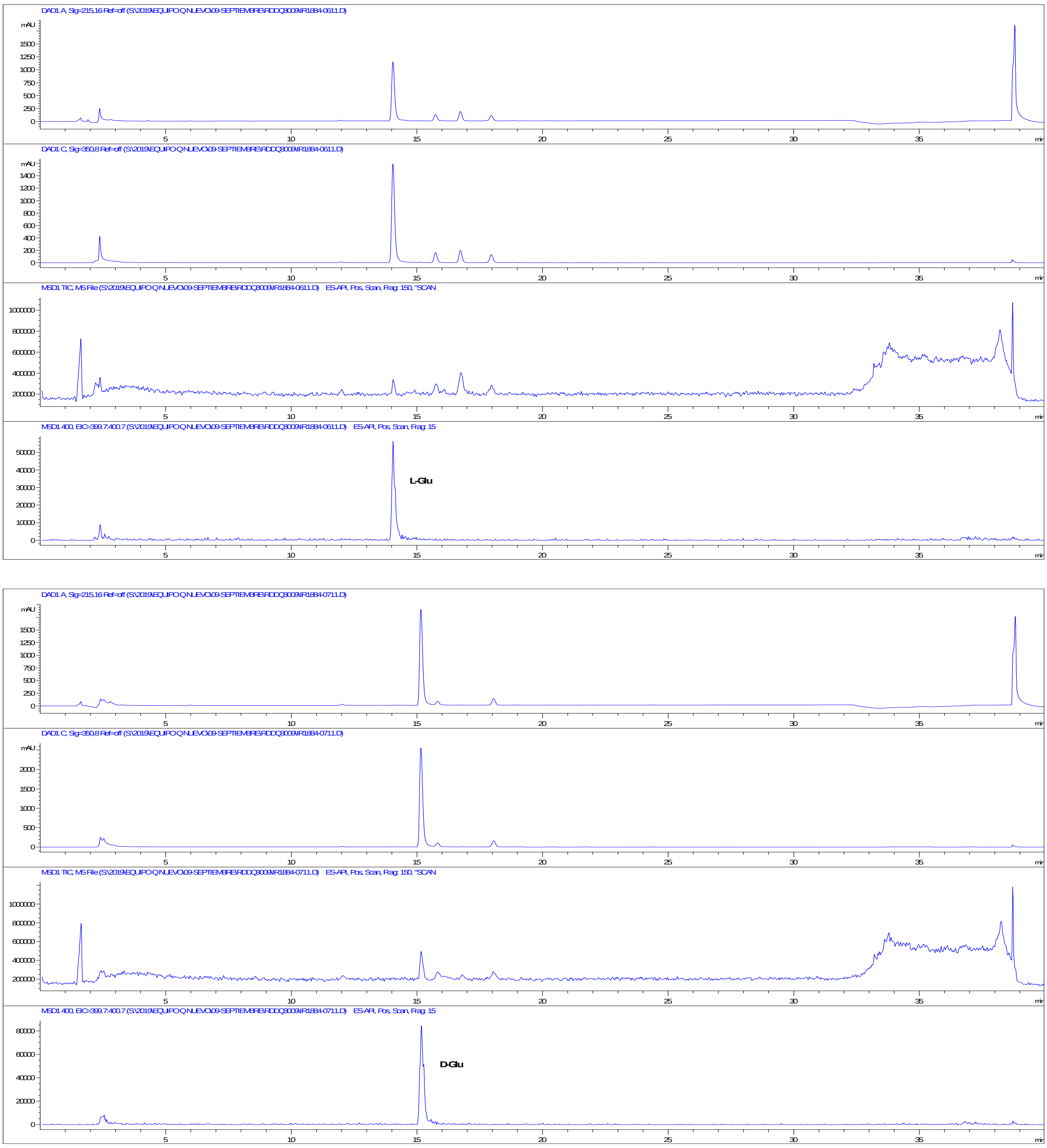

Figure S38 Marfey of Seribunamide A (**2**). Glu standards
